# Synthesis of 53 tissue and cell line expression QTL datasets reveals master eQTLs

**DOI:** 10.1186/1471-2164-15-532

**Published:** 2014-06-27

**Authors:** Xiaoling Zhang, Hinco J Gierman, Daniel Levy, Andrew Plump, Radu Dobrin, Harald HH Goring, Joanne E Curran, Matthew P Johnson, John Blangero, Stuart K Kim, Christopher J O’Donnell, Valur Emilsson, Andrew D Johnson

**Affiliations:** Division of Intramural Research, National Heart, Lung and Blood Institute, Cardiovascular Epidemiology and Human Genomics Branch, The Framingham Heart Study, 73 Mt. Wayte Ave., Suite #2, Framingham, MA USA; Department of Developmental Biology, Stanford University School of Medicine, Stanford, CA 94305 USA; Sanofi Aventis Pharmaceuticals, Bridgewater, NJ 08807 USA; Johnson & Johnson Pharmaceutical Research and Development, Radnor, PA 19477 USA; Department of Genetics, Texas Biomedical Research Institute, San Antonio, TX 78227 USA; Division of Cardiology, Massachusetts General Hospital, Boston, MA 02114 USA; Icelandic Heart Association, Kopavogur, Iceland

**Keywords:** eQTL, RNA, Gene expression, Genomics, Transcriptome, GWAS, Genome-wide, Tissue, Cis, Trans

## Abstract

**Background:**

Gene expression genetic studies in human tissues and cells identify *cis*- and *trans*-acting expression quantitative trait loci (eQTLs). These eQTLs provide insights into regulatory mechanisms underlying disease risk. However, few studies systematically characterized eQTL results across cell and tissues types. We synthesized eQTL results from >50 datasets, including new primary data from human brain, peripheral plaque and kidney samples, in order to discover features of human eQTLs.

**Results:**

We find a substantial number of robust *cis*-eQTLs and far fewer *trans*-eQTLs consistent across tissues. Analysis of 45 full human GWAS scans indicates eQTLs are enriched overall, and above nSNPs, among positive statistical signals in genetic mapping studies, and account for a significant fraction of the strongest human trait effects. Expression QTLs are enriched for gene centricity, higher population allele frequencies, in housekeeping genes, and for coincidence with regulatory features, though there is little evidence of 5′ or 3′ positional bias. Several regulatory categories are not enriched including microRNAs and their predicted binding sites and long, intergenic non-coding RNAs. Among the most tissue-ubiquitous *cis*-eQTLs, there is enrichment for genes involved in xenobiotic metabolism and mitochondrial function, suggesting these eQTLs may have adaptive origins. Several strong eQTLs (*CDK5RAP2, NBPF*s) coincide with regions of reported human lineage selection. The intersection of new kidney and plaque eQTLs with related GWAS suggest possible gene prioritization. For example, butyrophilins are now linked to arterial pathogenesis via multiple genetic and expression studies. Expression QTL and GWAS results are made available as a community resource through the NHLBI GRASP database [http://apps.nhlbi.nih.gov/grasp/].

**Conclusions:**

Expression QTLs inform the interpretation of human trait variability, and may account for a greater fraction of phenotypic variability than protein-coding variants. The synthesis of available tissue eQTL data highlights many strong *cis*-eQTLs that may have important biologic roles and could serve as positive controls in future studies. Our results indicate some strong tissue-ubiquitous eQTLs may have adaptive origins in humans. Efforts to expand the genetic, splicing and tissue coverage of known eQTLs will provide further insights into human gene regulation.

**Electronic supplementary material:**

The online version of this article (doi:10.1186/1471-2164-15-532) contains supplementary material, which is available to authorized users.

## Background

Genome-wide genetic analysis of gene expression [1, 2] identifies expression quantitative trait loci (eQTLs) which are mainly regulatory variants associated with *cis-* expression of nearby genes. Discovery of eQTLs may help elucidate the genetic mechanisms underlying natural variation in gene expression [3, 4]. Identifying these genetic variants may improve our understanding of molecular mechanisms of disease risk, and of potential drug targets. Human cross-tissue allele-specific expression studies indicate a significant fraction of genes are under genetic control by one or more alleles [5–7]. Strong eQTLs are often highly correlated with markers of disease and quantitative traits at loci identified in GWAS [8–13], suggesting that these eQTLs account for a significant fraction of human phenotypic variability. However, to date there are few attempts at characterizing cross-tissue eQTL datasets in a centralized manner.

Thus far, eQTL studies have analyzed gene expression traits measured primarily by DNA microarrays in liver [9, 14–16], multiple blood cell types [17–27], brain regions [24, 28–31], endothelial cells [32], stomach [9], skin [33], and adipose [9, 19]. Expression QTL effects are often partitioned into either *cis* or *trans-*acting effects, and few studies have thoroughly characterized *trans* eQTL associations, in part due to computational burden [34]. Furthermore, approaches to data collection and analysis of *cis* and *trans* eQTLs have been relatively non-uniform [34, 35]. Dimas et al. compared eQTLs discovered from 3 blood-related cell types [17], and found that only ~30% of eQTLs were directly shared across tissues. Later studies undertook multi-tissue comparisons of *cis-*eQTLs including lymphoblastoid cell lines (LCL) versus skin cells [33]; LCL, skin, and fat [36]; liver, omental, and subcutaneous adipose [9], and re-analysis of the Dimas et al. datasets with new methods [37]. Overall, these later studies found evidence for a high degree of sharing (~50-80%) of *cis*-eQTLs across tissues, while still indicating a significant minority of *cis*-eQTLs remain relatively tissue-specific. Prior studies compared at most 4 tissues and generally did not include external validation of signals or studies of *trans*-eQTLs. Thus, a rigorous comparison, across many tissues and populations with good statistical power remains relatively incomplete.

We sought to collect, standardize, and annotate a variety of eQTL results into a comprehensive central database in order to answer several basic research questions about eQTLs: 1) Are there master/housekeeping *cis* and *trans* eQTLs across tissues and what are their biologic functions? 2) What consistent *cis* and *trans*-eQTL patterns emerge across datasets including positional genomic location and overlap with regulatory annotations? 3) What genome-wide association (GWAS) variants converge with eQTL peaks? 4) Does integration of disparate eQTL data identify new *trans*-acting loci?

To address these questions we collected and analyzed available results from 53 eQTL population datasets. These 53 datasets represent analyses from 24 published manuscripts and 13 previously unpublished analyses reflecting >27 cell and tissue types. Most summary-level results are available for download as a subset of the NHLBI Genome-wide Repository of Associations between SNPs and Phenotypes (GRASPdb) [38].

## Results

### Characteristics of 53 gene expression GWAS (eQTL) datasets

The eQTL datasets (n = 53) collected included liver [9, 14–16], adipose tissues [9, 19], various brain tissues [24, 28–31] and blood lineage cells including whole blood [19, 20, 23, 25], lymphocytes [17, 21, 26], monocytes [24, 39], osteoblasts [22], fibroblasts [17] and Epstein-Barr transformed B-LCL [17, 18, 27]. Other tissues included kidney, stomach [9], skin [33] and peripheral artery plaque (see Table 1 for study summaries and [Additional file 1] for detailed characteristics). In some cases significant results beyond those originally reported were available via collaboration, otherwise the results reflected either new results from this paper or publicly available eQTL results that passed statistical correction thresholds defined by the original authors. The sample size varied widely across these studies (range n = 52-1,490, median n = 193, mean n = 311). Some of the 53 datasets reflected subgroup analyses (e.g., cases or controls, European or African ancestry). After common annotation of all datasets, dataset sample size showed modest logarithmic fit with the number of *cis*-eGenes identified (r^2^ = 0.45) and less so with *trans*-eGenes (r^2^ = 0.24) [Additional file 1]. This suggests many prior studies may have been underpowered but signal saturation may be approached with several thousand samples.

**Table 1:** **Summary of 53 eQTL datasets, their origins and original reported parameters**

Author (PMID)	Tissues (Sample size)	*cis*analysis	*trans*analysis	Imputation (SNPs tested)*	Genes analyzed
*Brain tissues*					
**Emilsson (23622250)** ^**†**^	DLPFC, VC, CR versus: All samples (n = 742), Alzheimer’s (n = 376), Huntington’s (n = 193)^†^, Normal (n = 173)	<1 Mb	Yes (diff. chr)	No (838,958)	39,579
**Kleinman (22031444)**	PFC_EA + AA + others (n = 269), PFC_AA (n = 147), PFC_EA (n = 112)	n/a	Yes (all)	No (625,439)	30,176
**Liu (20351726)**	PFC (n = 127)	<1 Mb	Yes	No (366,140)	6,968
**Webster (19361613)**	Cortex (n = 364), Cortex:Alzh (n = 176)	<1 Mb	Yes (≥1 Mb)	No (502,627)	24,357
**Myers (17982457)**	Cortex (n = 193)	<1 Mb	Yes (≥1 Mb)	No (366,140)	14,078
**Heinzen (19222302)**	Cortex (n = 93)	<100 kb	No	No (~550,000)	~22,000
**Gibbs (20485568)**	Temporal cortex (n = 144), Frontal cortex (n = 143), Cerebellum (n = 143), Pons (n = 142)	<1 Mb	Yes	Yes (~1,655,958)	~9,372^||^
*Blood tissues/cells*					
**Zeller (20502693)**	Monocytes (n = 1,490)	<1 Mb	Yes (≥1 Mb)	No (675,350)	12,808
**Fehrmann (21829388)**	Whole peripheral blood (n = 1,469)	≤250 kb	Yes (>5 Mb)	No (290,211)	19,609
**Goring (17873875)**	Lymphocytes (n = 1,240)	≤1 Mb	Yes	No (~500,000)	18,519
**Dixon (17873877)**	LCL (n ~ 400)	<100 kb	Yes (diff. chr)	No (408,273)	20,599
**Stranger (17873874)**	LCL (n = 210)	≤1 Mb	Yes (>1 Mb)	Yes (2.2 million)	13,643
**Murphy (20833654)**	CD4 + lymph (n = 200)	<50 kb	No	No (516,512)	19,904
**Idaghdour (19966804)**	Leukocytes (n = 194)	<50 kb	Yes (diff. chr)	No (516,972)	16,738
**Emilsson (18344981)**	Blood (n = 150)	<1 Mb	Yes (≥1 Mb)	No (317,503)	20,210
**Heap (19128478)**	PaxGene whole blood (n = 110)	<250 kb	No	No (257,013)	19,867
**Grundberg (19654370)**	Osteoblasts (n = 95)	<250 kb	Yes (diff. chr)	No (383,547)	18,144
**Dimas (19644074)**	Tcells (n = 85), Fibroblasts (n = 85), LCL (n = 85)	<1 Mb	No	No (394,651)	17,945
**Heinzen (19222302)**	PBMC (n = 80)	<100 kb	No	No (~550,000)	~22,000
*Other tissues/cells*					
**Greenawalt (21602305)**	Liver (n = 651), Subcutaneous Adipose (n = 701), Omentum (n = 848), Stomach (n = 118)	<1 Mb	Yes (>1 Mb)	No (~650,000)	39,303
**Schadt (18462017)**	Liver (n = 427)	<1 Mb	Yes (≥1 Mb)	No (782,476)	34,266
**Innocenti (21637794)**	Liver (n = 206), Liver (n = 60)	<250 kb	Yes^‡^	HapMap (rel.27)	14,703^||^
**Schroder (22006096)**	Liver (n = 149)	<1 Mb	Yes (>1 Mb)	No (299,352)	15,439
**Kim** ^**†**^	Kidney (cortex) (n = 81)	<1 Mb	No	No (906,600)	44,692
**Emilsson** ^**†**^	Peripheral artery plaque (n = 202)	<1 Mb	Yes (>1 Mb)	No (224,698)	37,582
**Emilsson (18344981)**	Subcutaneous Adipose (n = 150)	<1 Mb	Yes (≥1 Mb)	No (317,503)	20,210
**Ding (21129726)**	Normal Skin (n = 57), Psoriasis Lesional Skin (n = 53), Psoriasis UninvolvedSkin (n = 53)	<1 Mb	No	HapMap(rel.21)	~54,000
**Kompass (21226949)**	Endometrial Tumor (n = 52)	5 Mb	Yes (>5 Mb)	No (68,523)	8,543

“n/a” = not applicable. *Number of SNPs reported as being tested when specified. ^†^dataset which has not previously been published separately. ^‡^no *trans*-eQTL results given in the publication. ^||^# of snps and/or genes varied among datasets in this paper. The maximum is given. kb = kilobase. Mb = megabase. PBMC = peripheral blood mononuclear cells. LCL = Epstein-Barr transformed B-lymphoblastoid cell line. PFC = prefrontal cortex. DLPFC = dorso-lateral prefrontal cortex. VC = visual cortex. CR = cerebellum.

Genotyping and gene expression arrays across the datasets were heterogeneous (Table 1). Genotyping assays included Affymetrix (500 K, 6.0), Illumina (100 K, 300 K, 550 K, 610 Kquad, 650 K) and Perlegen SNP arrays (300 K, 438 K). Only a small proportion of datasets (n = 10, 18.9%) included imputed SNP analysis. Expression assays included custom arrays, Affymetrix (Human ST 1.0 exon, U133 plus A/B/2.0), and Illumina (WG-6 v1, WG-6 v3, HumanRefSeq-8 v2, HT12) arrays, with a mean of 20,246 RNAs interrogated across unique studies. Thus, these analyses primarily reflected mRNA expression of protein-coding genes, with few splice-specific analyses [24]. The datasets utilized different criteria for reporting significant results, including different multiple test correction thresholds and distance thresholds for defining *cis-acting* eQTLs (range = 100 kb to 5 Mb). As a result of these combined factors, as well as varying statistical power, whether *trans* analysis was conducted, and the extent of disclosed results, there were a broad range of significant eQTLs defined by the studies (range n = 33–22,473).

### Frequency of eGenes and eQTLs across 53 datasets after common annotation

**Figure 1:**
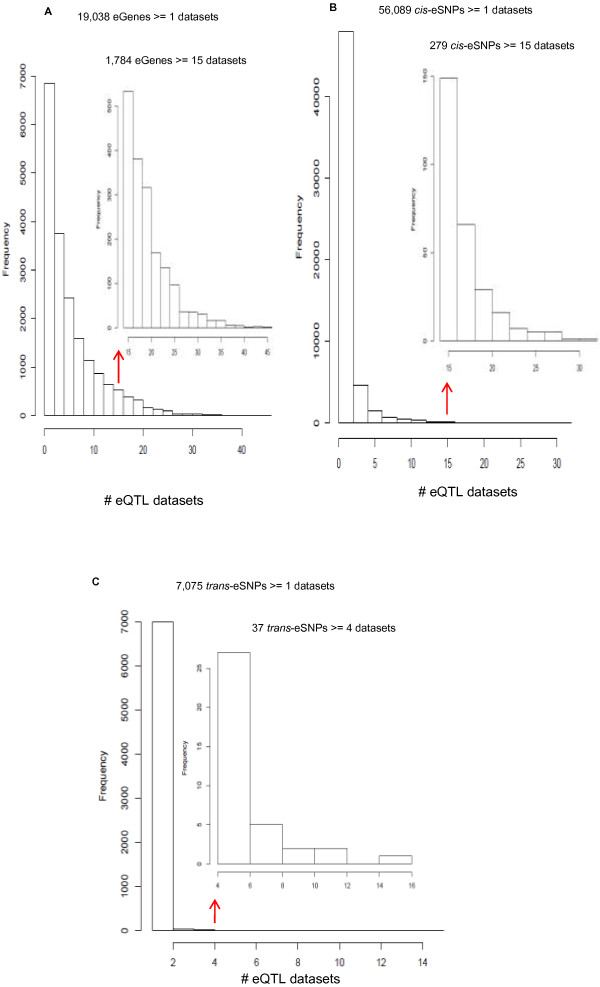
**Frequency of eGenes and eQTLs across 53 datasets. A**: Distribution of the occurrence of 19,038 unique eGenes across all 53 eQTL datasets. Inset: histogram of 1,784 genes found in > =15 eQTL datasets. **B**: Distribution of the occurrence of 56,089 unique, best *cis*-eQTLs across all 53 eQTL datasets. Inset: Histogram of 279 *cis*-eQTLs found in > =15 eQTL datasets. **C**: Distribution of the occurrence of 7,075 unique and best *trans*-eQTLs across all 53 eQTL datasets. Inset: Histogram of 37 *trans*-eQTLs found in ≥ 4 eQTL datasets. For each *trans*-eQTL, all proxy SNPs in perfect linkage disequilibrium (r^^2^ = 1 in CEU) are also included [42].

A total of 19,444 eGenes mapped directly to NCBI RefSeq gene symbols (n = 17,294) or RefSeq gene aliases (n = 2,150) [Additional file 2]. The majority of both eGenes and eQTLs were reported in only one dataset (Figure 1), which may reflect false positives, tissue-specific results, or a lack of statistical power, and SNP and/or transcript coverage differences across studies. Nevertheless, 1,784 eGenes were found in ≥30% of the datasets (n ≥ 15 datasets) (Figure 1A).

A total of 419,796 eQTLs passed at least nominal statistical correction thresholds in the 53 original sources. These included redundant eQTLs in relatively high linkage disequilibrium (LD) in some datasets. We retained the most significant eQTL for each eGene *within each dataset* yielding 116,563 “best” eQTLs from the constituent datasets. We mapped all best eQTLs in a common genome build (hg18) and applied a uniform distance threshold (500 kb) across all 53 datasets to define *cis* and *trans*-acting variants, finding 106,083 *cis*-eQTL-eGene associations (91%) and 10,480 *trans*-eQTL-eGene associations (9%). On average, each eGene is associated with 1.8 eQTLs. For 62,872 unique best eQTLs *across datasets*, 279 *cis* eQTLs are found in ≥30% of the datasets (N ≥ 15) (Figure 1B), while only 37 SNPs are *trans*-associated with eGenes in ≥ 4 datasets (Figure 1C).

### Master eQTLs with strong *cis*genetic influences across tissues

**Figure 2:**
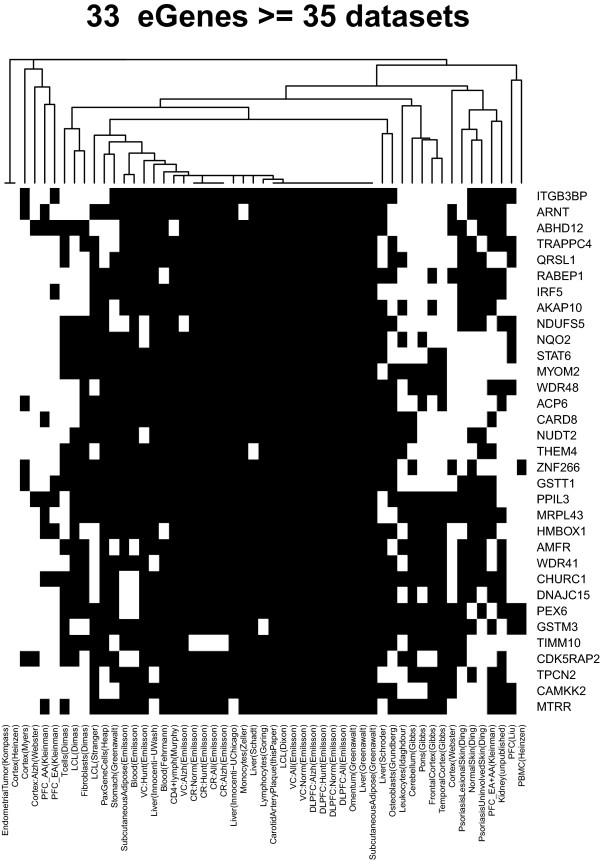
**Hierarchical clustering shows robust eGenes with strong genetic influences across a majority of studies.** eGenes present in >70% of datasets (>35/53 datasets). Individual datasets are indicated at bottom with eGenes listed to the right. Presence (black) or absence (white) of eGenes as eQTLs within individual datasets is shown.

To assess the most ubiquitous eQTLs, we examined 33 eGenes whose expression was significantly affected by SNPs in ~70% of datasets (n ≥ 35) and performed unsupervised hierarchical clustering (Figure 2). Several eGenes demonstrated strong genetic influences in more than 80% of datasets (n ≥ 42), including *PEX6, GSTM3, PPIL3, MRPL43, and CHURC1*. When compared against results from the GTeX (Genotype-Tissue Expression) project portal [40], 30 of these 33 eGenes had significant *cis*-eQTL in 2 or more of 9 independent tissues analyzed in that project (Table 2). The SNPs in Table 2 were checked for potential polymorphism in probe effects using PiPmaker [41]. None of the SNPs listed were found to directly overlap probes. Six of the SNPs had perfect proxy SNPs (r^2^ = 1.0) that overlapped one or more Affymetrix or Illumina probes (*ACP6, ARNT, ITGB3BP, GSTM3, NDUFS5, THEM4*), indicating a small minority of these widespread *cis*-eQTLs may be influenced by SNP in probe effects.

**Table 2:** **Most frequently occurring** *cis* **-eGenes across all datasets**

eGene	Datasets	Best eQTL, [#datasets], fxn*	Lowest P ^†^	CEU MAF	GTeX results‡	Most common eQTL, [#datasets], fxn*
*CHURC1*	43	rs10144942, [1]	1E-322	0.175	Y (9/9)	rs7143432, [29], 1.9 kb upstream
*PEX6*	43	rs2274517, [5], intron	1E-322	0.450	Y (9/9)	rs2395943, [26], intron
*PPIL3*	43	rs10167387, [2], intron	1.87E-292	0.225	Y (9/9)	rs7606251, [16], intron
*GSTM3*	42	rs10735234, [12]	1.10E-156	0.458	Y (9/9)	rs11101992, [13]
*MRPL43*	42	rs2863095, [25]	7.20E-120	0.208	Y (3/9)	<best eSNP, [25]
*GSTT1*	40	rs5760176, [1]	2.6E-317	0.375	Y (9/9)	rs4822458, [17]
*WDR41*	39	rs335628, [6], intron	1E-322	0.158	Y (5/9)	rs441102, [27], intron
*AMFR*	39	rs4924, [11], 3′UTR	9.80E-198	0.467	Y (3/9)	rs2440468, [12], intron
*ZNF266*	39	rs6512121, [14], intron	2.90E-183	0.483	Y (9/9)	<best eSNP, [14], intron
*HMBOX1*	39	rs8180944, [21], intron	1.53E-75	0.275	N (0/9)	<best eSNP, [21], intron
*DNAJC15*	38	rs17553846, [3], intron	6.11E-181	0.233	Y (9/9)	rs11617079, [19], nSNP
*MTRR*	38	rs3776455, [2], intron	2.60E-170	0.375	Y (2/9)	rs162036, [19], nSNP
*WDR48*	38	rs1274958, [3], nSNP	4.50E-142	0.258	Y (2/9)	rs12636980, [19], intron
*MYOM2*	38	rs9314455, [1]	8.40E-127	0.392	Y (6/9)	rs12681998, [9], intron
*CDK5RAP2*	37	rs3780674, [10], intron^a^	2.10E-172	0.092	N (0/9)	rs10125592, [18], intron^a^
*ABHD12*	37	rs2482911, [9], intron^b^	1.16E-104	0.417	Y (4/9)	<best eSNP, [9], intron^b^
*RABEP1*	37	rs11078559, [14], intron	4.01E-103	0.417	Y (4/9)	<best eSNP, [14], intron
*NUDT2*	36	rs10972063, [2], splice site	3.69E-182	0.108	Y (9/9)	rs10971957, [13]
*ACP6*	36	rs12119079, [12], intron	1.76E-84	0.325	Y (7/9)	<best eSNP, [12], intron
*ARNT*	36	rs11204726, [9]	2.80E-64	0.375	Y (3/9)	rs7412746, [13]
*AKAP10*	35	rs203462, [6], nSNP^c^	1.70E-132	0.408	Y (2/9)	rs397969, [8], 3.5 kb downstream^c^
*TPCN2*	35	rs4930265, [3], 3UTR^d^	5.50E-127	0.275	Y (3/9)	rs3750965, [16], nSNP^d^
*TRAPPC4*	35	rs11006, [11], 3UTR	1.10E-123	0.275	Y (9/9)	rs4938621, [16], intron
*ITGB3BP*	35	rs6697508, [15], intron	1.27E-114	0.283	Y (9/9)	<best eSNP, [15], intron
*QRSL1*	35	rs3101493, [22], 3UTR	7.90E-109	0.425	Y (8/9)	<best eSNP, [22], 3′UTR
*CAMKK2*	35	rs11065504, [7], intron	2.40E-107	0.300	Y (4/9)	rs3794207, [24], intron
*NDUFS5*	35	rs3768324, [8], intron	5.28E-48	0.375	Y (8/9)	rs10888650, [16]
*TIMM10*	34	rs2649667, [1], intron	5E-324	0.233	Y (8/9)	rs2848630, [18]
*STAT6*	34	rs324019, [4], intron	6.87E-198	0.392	Y (1/9)	rs841718, [24], intron
*CARD8*	34	rs1062808, [25], 3UTR	9.80E-198	0.292	Y (3/9)	<best eSNP, [25], 3′UTR
*NQO2*	34	rs1028612, [1]	6.12E-156	0.225	Y (9/9)	rs2071002, [16], nSNP
*THEM4*	34	rs13320, [25], 3UTR^e^	2.60E-93	0.383	Y (3/9)	<best eSNP, [25], 3′UTR^e^
*IRF5*	33	rs2172876, [1], intron^f^	1E-322	0.383	Y (3/9)	rs6965542, [12], intron^f^

*fxn = functional annotation of SNP; if no function is listed the SNP is intergenic. ^†^lowest eSNP p-value across all datasets where an eGene was reported. Results from the GRASP GWAS database for SNPs or those in perfect LD (r^2^ = 1): ^a^bone mineral density (P < 7E-7), ^b^alkaline phosphatase (P < 7E-10), ^c^platelet count (P < 2E-9), ^d^hair color (P < 3E-11), ^e^melanoma (P < 9E-11), ^f^anti-dsDNA in systemic lupus erythematosus (P < 2E-6). ‡GTeX (Genotype Tissue Expression Resource) results were queried for 9 tissues on August 6, 2013. Tissues queried included: adipose (subcutaneous), artery (tibial), blood, heart (left ventricle), lung, muscle (skeletal), nerve (tibial), skin (sun exposed), and thyroid.

These genes may represent housekeeping or master *cis-*eGenes, and could be useful positive controls in future studies. We next extended clustering to 248 high confidence eGenes found in ≥25 of our datasets [Additional file 3] and found eQTLs clustered by tissue type but were also greatly influenced by overlapping study samples. For example there was clustering of eQTLs from different brain anatomical sites derived from the same study samples, whereas an independent brain study which reported fewer eQTLs [28] was in a distinct cluster from the largest brain eQTL study [31]. Clustering was observed for three eQTL datasets in different blood cells that applied similarly stringent correction thresholds [17]. Pathway and ontology analysis of the 248 clustered *cis-*eQTLs revealed enrichment of genes involved in antigen processing and presentation and immune function, glutathione S-transferase activity, and mitochondrial function [Additional file 4].

We further characterized putative functional explanations for the 33 most ubiquitous *cis-*eGenes (Figure 2), for which gene symbols and basic functions are described in [Additional file 5]. All of the eQTL SNPs were common variants (the lowest MAF is 9% in CEU), and their signals were consistently large in effect (Table 2). The most frequent eQTL across datasets was often not the strongest eQTL but was highly correlated with the strongest eQTL, with a few exceptions (*NUDT2* pairwise r^2^ = 0.08, *NQO2* r^2^ = 0.11, *MYOM2* r^2^ = 0.17, *GSTM3* r^2^ = 0.20). These exceptions may reflect coverage differences across studies or allelic heterogeneity of functional variants at some loci. A functional characterization of all SNPs in Table 2 and their perfect proxies (r^2^ = 1.0 in 1000 Genomes phase I European samples [42]) indicates ~2/3 of loci had a perfectly correlated nonsynonymous SNP (nSNP), splice site SNP or UTR SNP, although functional interpretation was not always straightforward since there were multiple SNPs with putative function in some cases. We queried the SNPs in Table 2 against ENCODE regulatory features using RegulomeDB [43]. Most of the loci in Table 2 displayed one or more strong eQTL directly overlapping an ENCODE regulatory features (e.g., transcription factor binding site prediction, footprinting motif, chromatin structure features and/or protein binding (ChIP-seq feature)) [Additional file 6], suggesting many of them are likely functional regulatory variants. For example, rs3768324 was the strongest observed eQTL for *NDUFS5* in 8 datasets, overlapped abundant regulatory features including ChIP-seq peaks such as POL2, SRF, PAX5 and ELK4, and lay close to the transcription initiation site.

### Long-range *cis*and *trans*-chromosomal eQTL results

Thirty-seven eGenes had *trans*-association (>500 kb from the eGene to the eQTL, or the eQTL on a different chromosome) in 4 or more datasets (Table 3). The 4 dataset threshold was selected to reduce the effects of intra-study sample correlation since most eQTL publications contain ≤3 tissues from the same individuals. At least half of the 37 *trans* eGenes appeared to be long-range *cis* associations (>500 kb), and several appeared to be possible misinterpretations due to genes that map to multiple genomic locations. Among eGenes/eQTLs on different chromosomes, there were several known and replicated *trans*-eQTL loci, e.g., MHC class II region on chr6 [20], the *MAPT* region on chr17 [44, 45], and the *BCL11A*/*HBG* beta-globin interaction [20, 46]. A single chr12 SNP, rs10876864, exhibited strong *trans* associations with 9 targets on 9 different chromosomes, in 4 distinct tissues: liver, omental adipose, blood cells and prefrontal cortex. The same variant also showed strong *cis* associations with *RPS26*, and to a lesser degree, *SUOX* [Additional file 7], and was associated with vitiligo [47] *.* Notably, this variant is in high LD with rs11171739 (r^2^ = 0.86 in CEU) previously implicated in blood cell *cis* association with *RPS26* and *SUOX* and *trans* association with several targets, as well GWAS associations for Type I diabetes [20, 48]. Of the two variants, rs10876864 had strong *cis* and *trans* associations in a broader range of tissues, and aligned with histone signatures and >25 ChIP-seq binding signals [Additional file 6]. Additionally, rs10876864 is in perfect LD (r^2^ = 1 in CEU) with rs1131017, a SNP absent from all commercial genotyping arrays which is positioned near the transcription start site of *RPS26*. Many of the SNPs or proxies in Table 3 also overlapped with ENCODE regulatory features based on RegulomeDB queries [Additional file 6].

**Table 3:** *trans* **-eQTLs (>500 kb) observed in 4 or more datasets**

Chr	Pos (Mb)	Nearby gene(s) [#datasets], fxn*	*trans*eQTL(s) ^†^	eGene targets‡	eGene (distances)
1	143	NBPF ncRNAs [12], intron	rs10907360	Many targets	0.65-3.6 Mb
1	201	*PPP1R12B* [4], nSNP, splice site^a^	rs3881953,rs12734338,rs12743401	Many targets	other chr.
2	60	*BCL11A* [4], intron^b^	rs766432	*HBG1* [4] *, HBG2* [3]	other chr.
3	100	*CPOX* [4]	rs1461161,rs1675511	*DCAF12L1* [4]	other chr.
3	40	*ENTPD3* [5], intron	rs2371185	Many targets	other chr.
3	40	*ENTPD3, EIF1B* [4]	rs2123999,rs11717036	Many targets	other chr.
3	40	*ENTPD3, RPL14* [4],3′UTR, intron	rs9848083,rs4973898,rs11539046	Many targets	other chr.
3	42	*ULK4* [9], nSNP^c^	rs1052501,rs10212536,rs3934103	*CTNNB1* [9]	0.55-0.7 Mb
5	0.3	*SDHA* [4], intron	rs6869925,rs6878087	*SDHAP3* [4] *, KRT6B* [1]	other chr + cis
5	2	*SDHAP3* [4], intron, near TSS	rs7734561	*CEP72* [1] *, PDCD6* [3]	0.94-1.3 Mb
6	164	*PACRG* [9], 3′UTR	rs9306	*PARK2* [9]	0.58 Mb
6	31	MHC locus [6] ^d^	rs6457374,rs2247056	Many targets	other chr + cis
6	31	MHC locus [4] ^d^	rs2074488	Many targets	other chr + cis
6	33	MHC locus [7] ^d^	rs2395185,rs9268853,rs9268858, +1 other	Many targets	other chr.
7	74	*GTF2I* [4], intron	rs13238568	*GTF2IP1* [4]	0.52 Mb
10	48	*ZNF488* [4]	rs4342964	*ANXA8L2* [3] *, RP11-144G6.7* [1]	0.71-0.95 Mb
11	0.8	*RPLP2* [4], intron	rs10902222	*LRFN1* [3] *, HCN2* [1] *, FAM72B* [1]	other chr.
11	55	*TRIM48* [6]	rs10792252	*SPRYD5* [6]	0.78 Mb
12	55	*SUOX*, *IKZF4* [5] ^e^	rs10876864	Many targets	other chr.
16	68	*NFAT5* [4], intron	rs1064825	*AARS* [4]	0.56 Mb
17	34	*MRPL45* [4]	rs4329955,rs4514720	*TBC1D3B/C/G* [4]	1.8-2.2 Mb
17	40	*ENSG00000214447,CCDC103* [4], 5′UTR	rs2277616	*ITGA2B* [4]	0.51 Mb
17	41	*MAPT* [11], intron^f^	rs17651507,rs3785885,rs8079215	*ARL17A* [5] *, ARL17P1* [6] *, LRRC37A2* [5]	0.52-0.57 Mb
17	41	*CRHR1* [7], intron	rs12150547,rs2696425,rs418891, +46 others	Many targets	other chr.
17	41	*MAPT* [7], intron	rs1864325,rs17762165,rs17688922, +62 others	Many targets	other chr.
17	42	*MAPT,NSF* [7], synonymous, intron	rs199535,rs169201,rs199448, +2 others	Many targets	other chr.
17	42	*MAPT,KIAA1267* [4], intron	rs2532332,rs17659881,rs17660065, +6 others	Many targets	other chr.
17	42	*MAPT,KIAA1267* [4], intron	rs17660595,rs17563986,rs17649553, +53 others	Many targets	other chr.
19	22	*BC033373, ZNF99, ZNF486* + 6 other ZNFs [4], UTR	rs3817397,rs8112960,rs7254018	*ZNF595* [4] *, ZNF479* [2] *, ZNF679* [2] *, ZNF486* [1] *, ZNF99* [1]	other chr.
22	20	*PI4KA, CRKL* [4], intron	rs178058,rs5761386,rs4822700	*PI4KAP2* [3] *, POM121L10P* [1]	0.63-3.8 Mb

*Representative nearby genes are given. Number of datasets with ≥1 target eGene originating from this *trans*-eQTL locus are given in brackets. Functional annotation of *trans* eSNPs are given. †*trans* eSNPs were grouped within blocks of perfect linkage disequilibrium (r^2^ = 1). ‡Where there were limited targets the target eGenes are given with the number of datasets for each in brackets. For all loci including those with *Many targets* more detailed association information is found in Additional file 8. Results from the GRASP GWAS database for SNPs or those in perfect LD (r^2^ = 1): ^a^asthma (P < 2E-6), ^b^fetal hemoglobin (P < 2E-20), beta-thalassemia severity (P < 1E-10), ^c^blood pressure (P < 2E-7), multiple myeloma (P < 8E-9), ^d^many pleiotropic associations, ^e^type I diabetes (P < 2E-16), alopecia areata (P < 9E-8), adult asthma (P < 3E-6), ^f^progressive supranuclear palsy (P < 2E-120), Parkinson’s disease (P < 2E-16), primary biliary cirrhosis (P < 6E-6).

Our cross-dataset analysis also highlighted some interesting potential new *trans* signals. Target transcripts and tissue associations are fully described in [Additional file 8]. One set of correlated *trans* eQTLs on chr19p12 localized near zinc finger (ZNF) gene *ZNF429*, and was found within a large ZNF cluster including many genes. Notably the correlated eQTLs in this region were specifically associated in *trans* with the expression of zinc finger genes elsewhere in the genome-wide, including 4p16.3 (*ZNF595*), 7p11.2 (*ZNF479*), 7q11.21 (*ZNF679*), and within 19p12 (*ZNF99, ZNF486*). However, BLAT analysis [49] revealed that the chr4 and chr7 transcripts map with 83.5%-85.1% identity to the 19p12 region suggesting that gene homology and probe cross-hybridization could be responsible for the apparent *trans* associations. A SNP on chromosome 11, rs10902222, demonstrated strong *cis* associations mainly with *PNPLA2* and *RPLP2*, as well as *trans* associations with 3 different target regions (*LRFN1, HCN2, FAM27B*). A BLAT analysis of the SNP and the associated transcripts did not show homology indicating this may represent a new *trans*-eQTL locus [Additional file 9].

We additionally searched for distant eQTLs in 1 or more dataset with P < 5E-8 that overlapped long range regulatory interaction sites via ENCODE chromosome conformation capture carbon copy (5C) data [50]. Two SNPs had evidence for long-range interactions and eQTL association at this stringent threshold. Both SNPs were associated with expression in subcutaneous adipose (rs932562, P < 2.9E-22 for *WFDC2* (10.2 Mb away) [9]; rs1045001, P < 1.9E-8 for *RHBDL1* (0.62 Mb away) [19]) [Additional file 10]. However, the 5C interactions for both SNPs were more localized (up to 150 kb and 450 kb, respectively) than the eQTL associations (10.2 Mb and 6.6 Mb away) [Additional file 10]. Both variants also exhibit more localized, strong *cis* associations in other tissue datasets. This suggests medium-range regulatory effects of these variants, possibly corresponding to features identified by 5C, may in turn further influence longer range gene regulation megabases away.

### Significance of eQTLs relative to distance from eGenes

**Figure 3:**
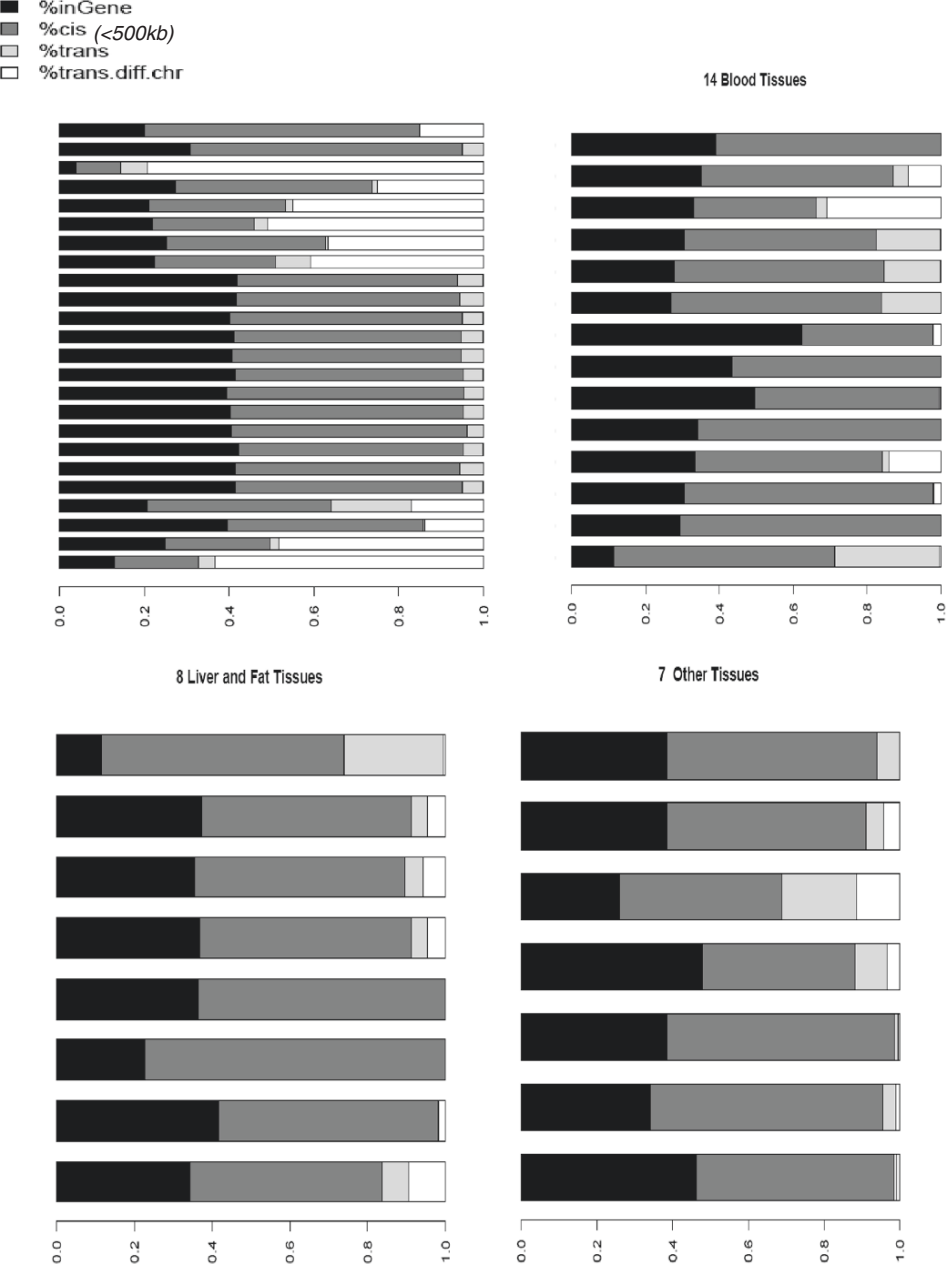
**eQTL-eGene distance distributions relative to datasets and tissue group.** Common SNP and transcript annotations were used to re-annotate all datasets and eQTL location categorized as: in the eGene, *cis* (≤500 kb from eGene), *trans* (>500 kb but on the same chromosome), *trans.diff.chr* (eQTL and eGene map to different chromosomes).

**Figure 4:**
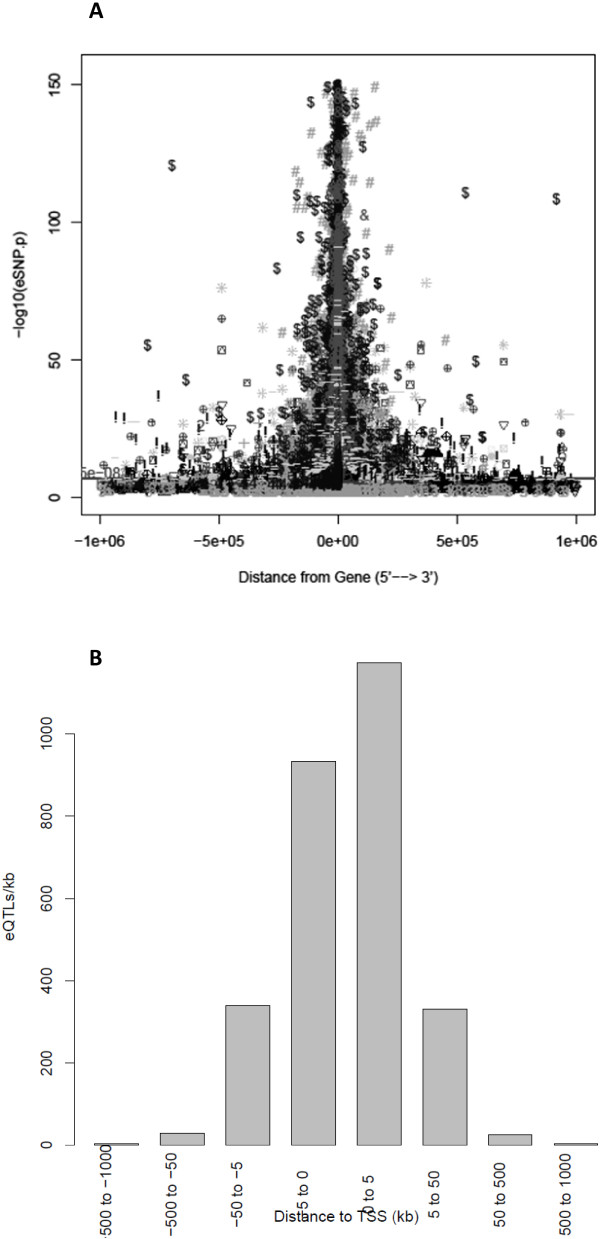
**Significance of eQTLs relative to distance from eGene boundaries. A**: 116,563 best eQTLs per eGene per dataset are shown across all 53 eQTL datasets. eQTLs located in their eGenes are plotted at 0 on the x-axis, otherwise the x-axis indicates distance of each eQTL to its eGene (from 5′: -1 Mb to 3′: +1 Mb). Not shown are 393 eQTLs with P < 1 × 10^-150^ which also display a highly central tendency. **B**: A histogram of the number of eQTLs per kb of distance from the 5′ transcription start sites (TSS) of eGenes.

Strength of eQTL signal correlated with the distance of the eQTL from its associated eGene boundary. Among 62,872 unique strongest *cis-* or *trans-*eQTLs, the majority of identified eQTL (89%) were located within *cis*-regions (*cis*-acting SNPs) (Figure 3), consistent with past reports [2]. There was a sharp drop in eQTL significance, as measured by P-values, near gene boundaries (median dataset kurtosis = 11) both up and downstream of eGene coding regions (Figure 4A), indicating eQTLs closer to their associated transcripts have higher significance. Individual dataset distributions split by 24 brain-related datasets, 14 blood, 5 liver, 3 fat and 7 other tissue datasets are shown in [Additional file 11]. Distributions of individual datasets were consistently kurtotic with only slight bias to the 5′ direction (median skewness = -0.032, mean SNP distance from gene = -1,356 bp). Results focused around 5′ transcription start site regions alone showed a strong central tendency within ±5 kb, with slight preference toward location in the downstream Exon 1 or 5′UTR direction (Figure 4B).

A minority of SNPs > 500 kb away from their associated eGenes were highly significant (0.5%, P < 1 × 10^-50^, 13.4% with P < 5e-8) (Figure 4A). Nonetheless, there were 7,075 significant eQTLs that are >500 kb distant from their associated eGene. The relative proportions of SNPs mapping within genes they are associated with, *cis* (1 bp-500 kb), *trans* (same chromosome >500 kb) and *trans* (different chromosome) is shown in Figure 3. Comparison across major tissue groups indicated an enrichment of *trans* (different chromosome) results in brain eQTLs relative to other tissue types (e.g., P < 0.002 relative to blood eQTLs).

### Enrichment of eQTLs within regulatory, selection and chromosomal features

To understand the spectrum of potential *cis* and *trans*-*acting* regulatory mechanisms across the human genome, we examined functional mapping of eQTLs to regulatory features from a variety of sources. A total of 62,872 unique best eQTLs were aligned against 22 regulatory feature datasets. Binomial tests indicated that these unique best eQTLs are localized within several regulatory features in the genome more than expected by chance (P < 0.01 for 14 out of a total of 22 regulatory features) shown in Table 4. Many of these features tend to co-localize closely to coding gene regions so overlaps may be expected based on the gene-centric tendency of eQTLs to associated eGenes. After adjustment for a variety of features, *cis*-eQTLs were most abundant (in order) on chromosomes 22, 21, 6, 20, 10 and 19, and least abundant (in order) on chromosomes Y, X, 7 and 3 [Additional file 12].

**Table 4:** **eQTLs compared to human genome regulatory features**.

Genome regulatory track	Nucleotides per track	Probability*	Expected overlaps	Observed overlaps	Obs:Exp	P-value
**ORegAnno**	11,265,267	0.00366	230	744	3.24	1.73E-159
**Functional RNAs**	107,202	3.48E-05	2.19	7	3.2	0.00725
**Gm12892V2.narrowPeak**	80,820,229	0.0262	1,650	4,610	2.79	<1E-308
**Gm12891V2.narrowPeak**	84,650,075	0.0275	1,730	4,680	2.71	<1E-308
**ENCODE H3k4me3**	120,458,965	0.0391	2,460	6,500	2.64	<1E-308
**Gm12878V3.narrowPeak**	43,937,796	0.0143	897	2,260	2.52	<1E-308
**ENCODE H3k27ac**	125,879,335	0.0409	2,570	6,540	2.55	<1E-308
**ENCODE H3k4me1**	242,340,600	0.0787	4,950	11,300	2.28	<1E-308
**Patrocles (miRNA database)**	3,375,454	0.0011	68.9	153	2.22	1.78E-18
**ENCODE H3k36me3**	631,024,019	0.205	12,900	28,200	2.19	<1E-308
**ENCODE CTCF**	44,516,245	0.0145	909	1,900	2.1	1.97E-185
**ENCODE 5C interactions** ^**†**^	10,484,463	0.34	214	510	2.38	8.80E-130
**CpG islands**	21,575,631	0.007	440	817	1.86	1.84E-58
**Conserved TFBS**	1,602,974	0.00052	32.7	54	1.65	4.00E-04
**miRbase (v.13)**	63,451	2.06E-05	1.3	2	1.54	0.371
**TargetScan**	354,030	0.000115	7.23	11	1.52	0.115
**ENCODE H3k27me3**	1,136,357,520	0.369	23,200	24,700	1.07	1.02E-37
**Vista Enhancers**	1,052,004	0.000342	21.5	16	0.745	0.906
**lincRNAs**	127,119,148	0.04	2,595	1,541	0.59	1
**IHS sites (Z-score > 3)**	2,275,923	0.000739	46.5	24	0.52	1
**F**_**ST**_ **sites (Z-score > 3)**	4,088,207	0.00133	83.4	41	0.49	1
**PolymiRTS predicted miRNA binding sites**	11,265,267	0.00366	230	1	0.00435	1

*Probabilities determined based on the fraction of the human genome covered by the feature track (human genome length = 3,080,436,451) and the total unique eSNP positions (n = 62,872). P-values are for binomial tests for enrichment of observed over expected. All ENCODE feature tracks are for lymphoblastoid cell lines and all are for sample GM12878 except where indicated. ^†^ENCODE 5C long range interactions targeted ~1% of the genome this coverage and expectations were derived based on this proportion, and 1% of the unique eSNP positions. TFBS = transcription factor binding sites. miRNA = microRNA. lincRNA = long, intergenic non-coding RNA. IHS = integrated haplotype score. F_ST_ = Fixation index.

### Housekeeping genes are more often eQTLs

**Figure 5:**
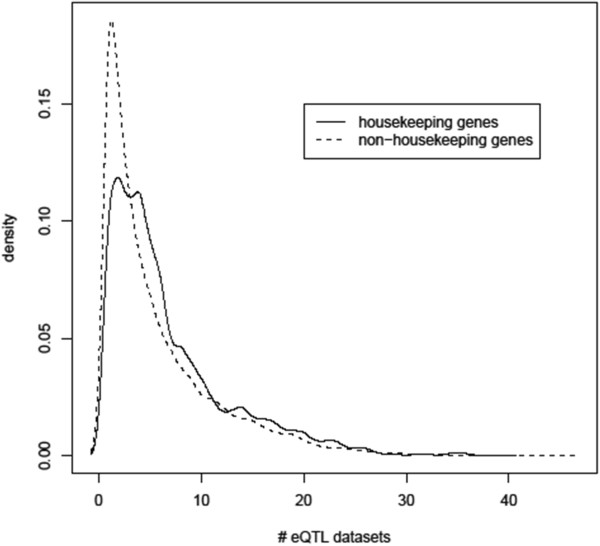
**Housekeeping genes are over-represented among eGenes common to many tissue datasets.** A density plot of eGenes that are housekeeping versus non-housekeeping genes (as defined by [51]) across datasets. The eGene distributions differ significantly (P < 1.12 × 10^-11^).

When a gene is expressed in multiple tissues or cells at relatively constant levels, regulatory control may be common across the tissues. To investigate the relationship between housekeeping and non-housekeeping eGenes we categorized them based on a previous analysis of publicly available expression data in 18 human tissues [51]. Out of 19,038 unique eGenes in our study, 2,207 were defined as housekeeping genes and 16,831 as non-housekeeping genes. A density plot of housekeeping eGenes showed they are more overrepresented in the right tail of distribution than non-housekeeping eGenes (Figure 5, P < 1.12 × 10^-11^, Student’s t-test).

### Expression QTL concordance with GWAS peak signals

Expression QTLs from the current study were compared against the NHGRI GWAS catalog. Since many eQTL studies did not conduct imputation we also assessed the overlap with LD perfect proxies for the GWAS catalog SNPs (r^2^ = 1) [42]. Among 8,845 unique GWAS SNPs, 926 were directly found among 62,872 unique best eQTLs (~10.5% overlap) [Additional file 13]. For these 926 common SNPs, there was significant positive correlation in strength of signal (assessed by P-values) between reported eQTL and trait GWAS associations (Spearman’s P = 2.75 × 10^-26^, [Additional file 14]. When LD partners (r^2^ = 1) are incorporated ~22% of GWAS catalog signals corresponded to a best eQTL association in our database. The NHGRI catalog was limited to selected top results, thus we further compared both eQTL and nSNP distributions within the test distributions of 45 full GWA trait scans for a variety of human disease, dichotomous and quantitative traits. For most GWA scans (n = 38/45) we found significant enrichment of eQTL SNPs among significant GWA results across the full test statistic distributions [Additional file 15]. Non-synonymous SNPs showed less enrichment (n = 13) and were significantly depleted in some scans (n = 2). This pattern persisted at the significant tail of the distribution (limiting to GWAS P < 1E-2) where 25 of 45 GWA were enriched for eQTL SNPs whereas only 3 GWA showed enrichment for nSNPs and 11 indicated depletion of nSNPs among significant results.

### Novel plaque and kidney eQTLs linked to GWAS results

To our knowledge, the plaque and kidney eQTLs in this study are the first reports for these tissues. We queried eQTLs from these tissues against non-anthropomorphic GWAS results in the GRASP database. Results are reported for kidney in [Additional file 16] and peripheral artery plaque in [Additional file 17]. Serum creatinine and creatinine estimated glomerular filtration rate are associated with rs835223 [52], which is also associated with *DAB2* expression levels in kidney here (P < 1.4E-5). Antibodies in systemic lupus erythematosus (SLE) accumulate in tissues including the glomeruli of kidney. SNP rs7808907 is associated with *IRF5* expression levels in kidney (P < 3.9E-13) and was previously associated anti-double stranded DNA autoantibody status in SLE [53].

SNP rs2133189 was previously linked to coronary artery disease (CAD) susceptibility [54] and is strongly linked here to peripheral artery plaque expression levels of *AIDA* (P < 2.1E-20). Other peripheral plaque eQTLs for SNPs previously linked to CAD or myocardial infarction include *BTN3A1* (rs6929846 eQTL P < 2.8E-07, myocardial infarction P < 3.5E-24 [55]), *ZNF344* (rs4803750 eQTL P < 3.8E-05, atherogenic dyslipidemia P < 1.3E-33 [56]), *NBEAL1* (rs6725887 eQTL P < 2.7E-06, CAD P < 1.1E-09 [57]), *ENST00000318084* (rs10764881 eQTL P < 2.7E-05, CAD P < 1.4E-09 [58]).

## Discussion

In this study, we systematically characterized and annotated eQTL results from 53 genome-wide gene expression GWAS datasets. Overall 19,038 genes had at least one eQTL significantly associated with their expression. Even if a substantial proportion of these represent false discoveries, a large proportion of human genes seem to have common genetic influences on their expression level, consistent with prior surveys using sensitive allelic specific expression methods [6, 59]. Given that few studies have explicitly assessed genome-wide genetic effects on splicing and alternate isoforms in human tissues there likely remain many additional genetic effects on expression to be discovered. Regional *cis*-eQTLs predominate genome-wide over *trans-*eQTLs, though limitations in statistical and computational power have hampered *trans*-eQTL discovery and validation.

We identified many *cis* and several *trans*-eQTLs that have evidence for consistent association across more than one study or tissue. These human master *cis-* and *trans-*eQTLs may serve as potential positive controls in future studies and may reveal important aspects of regulatory interactions and human biology and evolution. Furthermore, future researchers searching for and claiming tissue-specific eQTLs could screen their results against the results we collated and deposited in the GRASP database to ensure there is no prior evidence in other tissues. The strong effects and common allele frequencies of these variants may also make them useful in sample forensics in expression-based research [60].

Ubiquitous *cis-*eQTLs were enriched for housekeeping genes consistent with a prior study [61] and for several biological categories including antigen presentation, mitochondrial function and S-glutathione transferase activity. We speculate these *strong* cis-eQTLs of common allele frequency could represent beneficial alleles arisen in human evolution that may enhance immune function, mitochondrial function and xenobiotic metabolism. Glutathione S-transferases are responsible for detoxification of many compounds and five such transcripts were found among strong *cis*-eQTLs (1p13.3: *GSTM1, GSTM3, GSTM4,* 22q11.23: *GSTT1,* 10q25.1: *GSTO2).* GSTM1 *and* GSTT1 have previously been reported to be subject to copy number variation influencing gene expression [62, 63]. Results integrated across studies here reveal other members of the glutathione are subject to strong genetic regulation. Mitochondrial-associated transcripts were significantly enriched making up 12.1% of the *cis*-eGenes present in ≥25 datasets. These include genes that encode mitochondrial proteins involved in the electron transport chain and ATP synthesis (*NDUFS5, COX7A2L, ATP5S*), membrane functions (*AKAP10, FECH, SURF1, TIMM10*), transport (*SLC25A16*), and mitochondrial protein synthesis (*MRPL19, MRPL21, MRPL43*). While overall eQTL results were not enriched for overlap with selection features as defined by integrated haplotype scores or fixation index (F_ST_), several of the master eQTL regions correspond with regions identified as containing human lineage-specific events [64]. These include *CDK5RAP2* which appears to be under positive selection and may be involved in increased human brain size [65, 66], and the *SRGAP2* and *NBPF* gene cluster on chromosome 1 which demonstrates human lineage copy number increases and is suspected to play a role in increased neuronal branching in development [67–69].

We examined positional effects of eQTLs with respect to associated transcripts, regulatory features and across chromosomes. The strongest eQTLs cluster around their associated gene transcript regions, a pattern that appears universal across tissues and datasets, and is consistent with prior reports considering smaller numbers of tissues (e.g., [17]). A variety of regulatory features overlap eQTLs more than expected by chance, as others have also reported [70, 71]. This is partially expected given gene co-centricity of these features and eQTLs. Features that lacked significant enrichment among eQTLs included microRNA coding regions and targets, human enhancer regions and non-coding RNAs. Thus, these features may account for a smaller proportion of functional genetic regulation of gene expression. This may be a property of more distant location from coding genes (i.e., enhancers, non-coding RNAs) but could also suggest less tolerance of functional variation in these features. Analysis across chromosomes reveals that chromosomes 21 and 22, in particular, display higher rates of *cis*-eQTLs after adjusting for a number of factors including gene number, coding length and number of variants. Notably, chromosomes 21 and 22 have been subject to major shifts in primate and human evolution [72].

Unlike the abundant *cis*-eQTLs, there appear to be few *trans*-eQTL hotspots across the genome. Many studies have chosen not to calculate long range *cis-* or *trans*-eQTL effects. Furthermore, given the large multiple testing burden discriminating true positives from false positives is challenging, particularly with limited statistical power, and if replication is not attempted. Homologous transcript mapping and cross-hybridization artifacts may also confound *trans*-eQTL discovery in some cases. Nonetheless, a few *trans*-acting regions have emerged with consistent evidence across a number of studies, including the HLA region (6p21.32), *ARHGEF3* (3p14.3), the *MAPT* region (17q21.31), *HBG* (11p15.4), *SUOX-IKZF4-RPS26* (12q13.2), and now *RPLP2-PNPLA2* (11p15.5). Most of these regions have been implicated by human disease GWAS. Combining data across studies and tissues may help resolve mechanisms, key targets, and the extent of targeted expression networks. For example, our study suggests that *RPS26-*associated variants may be the key *trans* regulators at 12q13.2. Data from subcutaneous adipose included in the current study suggest rs4731702 near *KLF14* (7q32.3) is associated in *trans* with *SLC7A10* expression, which supports *SLC7A10* as an important *trans* adipose target associated with metabolic traits as previously suggested [73]. Greater sample sizes may be needed to find and validate more *trans*-eQTLs, or the application of other approaches such as analysis of co-expressed modules [48], multi-species studies or addition of functional screens.

Prior studies suggested enrichment of eQTLs among some full GWAS scans and among topmost significant results. Here we examined a greater number of tissue eQTLs and GWAS results. Among 45 full human GWAS scans of disease and non-disease traits, we observe a consistent pattern whereby there is enrichment of eQTLs above and beyond nonsynonymous SNPs, and across the significant tail of the statistical distributions. This suggests that eQTLs contribute to the multi-genic nature of many complex human traits and may account for a greater proportion of variance than protein-coding variation [74]. In an analysis focused on strongest GWAS results from the NHGRI catalog we observe significant correlation between the strength of signal for GWAS and expression traits. Concordant strongest GWAS and eQTL SNPs establish a conservative floor indicating ~10% of GWAS phenotype signals are likely directly attributable to genetic regulation of expression. The true proportion of functional regulatory variants is likely much higher given functional alleles in LD, and incomplete coverage in the available eQTL results for variants and human populations, alternative splicing, non-coding RNAs, and tissue-specific expression. Overall these results imply that eQTLs will remain a critical component in interpreting genetic associations and prioritizing replication candidates for a variety of traits.

The addition of new tissue eQTLs may continue to suggest new mechanisms or reinforce prior hypotheses for functional variants. Here we report the first human kidney and plaque eQTLs. Kidney eQTLs corresponded with several prior kidney-related GWAS findings. Several findings of peripheral plaque eQTLs were for variants previously associated in GWAS of coronary artery disease or myocardial infarction. Notably, a prior study reported rs6929846 to be associated with myocardial infarction in a Japanese GWAS sample and replicated the finding in a subsequent Japanese sample [55]. Yamada et al. also provided evidence for rs6929846 transcriptional effects on *BTN2A1* expression, and immunohistological positivity for BTN2A1 in human myocardial infarction lesions, and coronary endothelium, arterioles and capillaries [55]. Our study links the same SNP to expression levels of nearby *BTN3A1* in peripheral artery plaque (P < 2.8E-7). This locus contains 6 butyrophilin genes and 1 butyrophilin pseudogene. The combination of these results suggests butyrophilin genes may play roles in coronary artery disease pathogenesis, possibly through roles in antigen presentation and T cell stimulation [75].

Beyond limitations in the analysis of *trans-*eQTLs this study has several significant limitations. The full gene expression-SNP datasets are generally unavailable, so the current catalog is limited by significant results available from individual studies, and probe annotations are often missing limiting precise localization and assessment of potential probe artifacts. The specific studies are biased mainly toward more readily available tissues, including blood, B-lymphoblastoid cell lines and brain autopsy tissues. Studies were further biased by their non-uniform transcript and genetic content and statistical power. Overall these limitations suggest the current database would most likely be prone to false negatives, thus lack of association at a specific locus cannot be viewed as definitive.

The decrease in the cost of genome-wide genotyping, sequencing and expression profiling means that larger sample sizes are increasingly feasible for eQTL studies. Applying RNA sequencing to eQTL studies may increase discoveries particularly with regard to genetically regulated alternative splicing [3, 4]. While still in early stages, the study of additional RNA types such as long non-coding RNAs [76] and micro RNAs and their targets [77, 78] and corresponding tissue-specific QTLs is leading to new insights. Deeper profiling of eQTLs via dense imputation with a modern 1000 Genomes based genetic map should increase eQTLs and improve fine mapping as recently demonstrated [79]. Profiling a greater proportion of human tissues as undertaken by the GTex project should further aid in defining tissue-specific eQTLs [80]. These are important goals since eQTLs seem to account for a significant proportion of human phenotypic and disease variability. Many areas require further study *at the population level* including detailed probing of extensive tissue and cell types, and ascertainment of QTLs related to splicing [4, 24], RNA decay mechanisms [81], non-coding RNA [76, 82], and epigenetic mechanisms such as methylation [28, 83–85]. A deeper understanding of RNA-driven QTLs, whether *cis* or *trans,* tissue-specific or ubiquitous, coding or non-coding, splicing-, decay- or epigenetic-related may be critical to the interpretation of human phenotypic variability, in order to further disease risk prediction, understand causal mechanisms, and enable targeted therapies.

## Conclusions

Expression QTLs inform the interpretation of human trait variability, and may account for a greater fraction of phenotypic variability than protein-coding variants. Our analysis of >50 eQTL datasets, in a more extensive set of tissues than previously characterized, highlights the gene centricity of eQTLs and their overlap with regulatory features, as well as their strong enrichment in significant GWAS results for a wide variety of traits. Novel *trans*-eQTLs are suggested by our study but overall their identification remains challenging. Using new eQTL data from kidney and peripheral plaque we note intersections with GWAS for renal and arterial disease associations which may suggest causal genes or functional mechanisms. This large-scale synthesis of available tissue eQTL data identifies many strong and relatively ubiquitous *cis*-eQTLs that could serve as positive controls in future studies. Our results also suggest some of these common and strong tissue-ubiquitous eQTLs may have adaptive origins in humans. Efforts to expand the genetic, splicing and tissue coverage of known eQTLs will provide further insights into human gene regulation.

## Methods

### Ethics statement

Approvals for published eQTL studies are described in their original publications. New eQTL samples (kidney, peripheral artery plaque) described in conjunction with this study were collected with written informed consent and under institutional approvals. For the kidney eQTL study ethical approval for the study was obtained from the Stanford University Institutional Review Board (IRB protocol 3941). That study was conducted according to the principles expressed in the Declaration of Helsinki. Multi-institutional approvals for the collection of peripheral artery plaque tissue were previously described [86].

### Selection and collection of eQTL datasets

Many eQTL studies have been published in human and non-human species across a broad range of tissue and cell types. Early eQTL studies focused on the heritability and genetic basis of gene expression including several studies on lymphoblastoid cell lines used in the HapMap project. Several studies evaluated genetic variants related to drug response in cell lines. We focused our studies primarily on minimally altered human cells and tissues. Only one of the largest analyses of HapMap LCL samples was included here [27], and drug response, methylation, miRNA and non-human eQTL studies were excluded. Several published eQTL studies were not included since authors disclosed few results. Included studies, their citations and parameters are described in Table 1 and [Additional file 1]. The predominant tissue datasets are brain (n = 24 studies) and blood (n = 14), with other tissues including liver, adipose depots, kidney, skin, stomach and peripheral artery plaque. Previously unpublished data on kidney and peripheral artery plaque eQTLs are described in [Additional file 18]. Some previously published results were more extensively shared for the current analysis including liver, adipose and stomach [9], and lymphocytes [21].

### Unifying eQTL and eGene annotations into a cross-dataset database

The workflow of the complete analysis is delineated in [Additional file 19]. We define genes whose expression levels are significantly associated with SNPs as *eGenes*. The term does not explicitly imply a specific transcript isoform since this information is often indeterminable with available data, but is likely to reflect expression variation in dominant gene isoforms. We refer to SNPs associated significantly in combination with an *eGene* as *eQTLs* (expression QTL SNPs). After we removed duplicate entries in some datasets, we used custom programs to map remaining identifiers either directly to unique NCBI Entrez Gene IDs, or via alias identifiers for heterogeneous gene names, in order to create a harmonized eGene dataset for further analysis. Only the strongest eQTL was kept for each eGene in each study in most subsequent analyses. Unified genomic locations (see Method below) for each eGene and eQTL in hg18/b36 reference were used to recalculate eQTL-eGene distances and direction (5′/- or 3′/+), and this dataset was used for subsequent analysis.

### Filtering of low quality SNPs and unification of SNP genomic coordinates

Studies either reported no SNP coordinates, or reported them in hg18 or hg19 frameworks. We mapped all of the SNP rsIDs reported in 53 datasets to dbSNP130 and used dbSNP reference genome mappings to obtain uniform genomic position for SNPs in hg18/Build 36.3. We removed SNPs which mapped to >1 location, or to the pseudo-autosomal region. For SNPs not initially mapped by this approach we checked for alias SNP identifiers to link to dbSNP130, and used the alias IDs when available to complete mapping. In this manner the majority of eQTLs were mapped to a single genomic position with high confidence.

Genomic locations for each gene boundary were retrieved from NCBI RefSeq 56 (GRCh36.3 assembly) using hg18/b36 reference. If multiple transcripts/isoforms are transcribed from the same genomic locus/gene region the maximal union of boundaries was used. Data were retrieved using the biomaRt package [87], available through the Bioconductor repository [88]. eQTLs ≤ 500 kb from associated eGenes were defined as *cis*. Those eQTLs > 500 kb were defined as *trans*, and further segmented into those being *trans* on the same or different chromosomes.

### Summary of eGenes and eQTLs mapped to different categories

In total 419,796 eQTLs were reported from the 53 eQTL datasets. Among them, 359,268 eQTLs and their associated eGenes were mapped to RefSeq gene symbols or gene aliases, indicating both eQTL and eGene genomic positions in the RefSeq database. We selected the strongest eQTL per eGene per unique dataset yielding 116,563 best eQTLs (106,083 *cis* and 10,480 *trans* with the 500 kb threshold). Among these, there were 62,872 unique SNP identifiers that were the best eQTL in 1 or more dataset, for a total of 19,038 mapped eGenes.

### Unsupervised hierarchical clustering

Unsupervised hierarchical clustering was used to assess patterns of regulatory variants across different tissues and cell types. Initially a 19,038 × 53 data matrix was constructed. Given the sparse nature of the matrix (most eGenes are unique to 1 study), we generated clusters based on eGenes present in higher proportions of studies (n = 15-53). The heatmap function in R 2.11 was used to do clustering with the Disfun parameter set to binary.

### Comparison of eQTLs to NHGRI GWAS catalog

The NHGRI GWAS catalog (March-22-2013) was downloaded [89]. Expression SNPs strongly associated with the gene expression traits were cross-referenced with SNPs in the GWAS catalog. Two sets of eQTLs were compared (160,580 unique eQTLs and 62,872 unique *bes*t eQTLs) against two sets of SNPs derived from the GWAS catalog (8,845 unique SNPs and 40,573 unique SNPs plus those in tight LD (r^2 = 1 in CEU based on SNAP [42] queries)) yielding four pair-wise comparisons.

### Enrichment of eQTLs over protein-coding SNPs in full GWA trait scans

Full GWA trait scan statistics (n = 45 scans) were identified as part of the NHLBI GRASP database [38] and downloaded. Genomic lambda values were calculated relative to the null expectation for the full GWA distributions [90]. Likewise, lambda values were calculated within each GWAS for expression SNPs from the current study (n = 62,872 best eSNPs) and nSNPs (based on dbSNP annotation, n = 100,601). Further lambda values were calculated restricted to those GWAS results with P < 1E-2. The ratios for enrichment were determined by comparing lambda values of eQTLs versus non-eQTLs, and nSNPs versus non-nSNPs. Komologorov-Smirnoff tests were applied to test differences in the distributions under each criterion. Individual lead *cis*-eQTLs and *trans*-eQTLs were directly assessed for presence in the GRASP database containing results from among 1,390 GWAS studies.

### Comparison to human genome and regulatory features

We compared only the 62,872 *unique best* eQTLs to regulatory tracks. To take into account the different size of features (base pairs) reported by different tracks, for each regulatory track, the probability of any random base overlapping each track was calculated as the number of unique bases in each track divided by the total bases in the genome (3,080,436,451). Based on this probability, the expected number of overlaps between 62,872 single base position eQTLs and each track was computed. Binominal tests indicated whether observed overlaps were greater than expected by chance.

Regulatory tracks (B36 coordinates) were downloaded from the UCSC Genome Browser [91] or other sites. The 22 regulatory features include ENCODE histone modification sites, transcription factor and CTCF insulator sites in lymphoblastoid cell lines, ORegAnno (Open Regulatory Annotation) [92], predicted TFBS (UCSC conserved transcriptional factor binding sites), Vista Enhancers [93], human selection sites as determined by F_ST_ and IHS (integrated haplotype scores), human microRNAs (miRbase13*)* [94], TargetScan (predicted miRNA targets) [95], Patrocles (experimentally supported miRNA sites) [96], PolymiRTS (predicted SNP-miRNA binding sites) [97], UCSC functional RNAs (e.g., tRNA), UCSC CpG islands, long intergenic non-coding RNAs [98], and long-range 5C experiments in targeted ENCODE regions [50]. Specific top *cis-* and *trans*-eQTL SNPs were queried against ENCODE data using RegulomeDB [43].

The unique best *cis-*eQTLs were analyzed for differential representation by chromosomes. The total number of *cis-*eQTLs for each chromosome was divided by 4 distinct features to produce 4 rankings for enrichment: 1) total chromosome length (GRCh37.p11), 2) number of CCDS genes (release 11), 3) length of HuRef RNAs, and 4) number of HuRef variants. The chromosome rankings by the 4 metrics were averaged to produce an overall rank for over-representation of *cis*-eQTLs.

### Housekeeping gene analysis

Housekeeping transcripts were defined based on previous analysis of 18 human tissues [51]. Within our dataset 2,207 eGenes were designated as housekeeping genes and 16,831 as non-housekeeping genes. Frequencies of each eGene across dataset were calculated for housekeeping and non-housekeeping genes and compared by Student’s t-test.

## Availability of supporting data

The primary data for some of the eQTL studies is available in public repositories as described in the original reports. The summary level eQTL results data sets supporting the results of this article are largely available in the full download of the NHLBI Genome-wide Repository of Associations between SNPs and Phenotypes (GRASPdb) [Build 1.0, http://apps.nhlbi.nih.gov/grasp/] [99].

## Electronic supplementary material

Additional file 1: **eQTL dataset origins and descriptions.** eQTL dataset sources and information about sample sizes, total *cis* and *trans* eQTLs and eSNPs, SNP and expression platforms. (XLSX 14 KB)

Additional file 2: **Summary of all eQTLs and eGenes and their mapping and filtering.** Description of filtering steps and number of eQTLs, eSNPs and eGenes. (XLSX 10 KB)

Additional file 3: **Hierarchical clustering analysis of 248 eGenes found in ≥ 25/53 datasets used in pathway and ontology analyses.** Clustering diagram of eGenes found in ≥ 25 datasets. (DOC 128 KB)

Additional file 4: **Pathway and ontology analysis results for 248 most ubiquitous eGenes.** Significantly enriched gene categories among highly repeated eGenes across tissues. (XLSX 13 KB)

Additional file 5: **Full gene names and descriptions for 33 eGene significant in ≥35 datasets.** Full gene names and descriptions for 33 eGene significant in ≥35 datasets. (XLSX 13 KB)

Additional file 6: **Overlap of master-cis and trans-eQTLs with ENCODE regulatory features.** Intersection of master-cis and trans-eQTLs with ENCODE regulatory features (transcription factor position weight matrices, DNA footprinting motifs, chromatin structure, protein binding by chIP-seq) as determined with RegulomeDB queries. (XLSX 17 KB)

Additional file 7: **Trans-eQTL and cis-eQTL associations in chr12q13.2 region.** Trans-eQTL and cis-eQTL associations in chr12q13.2 region. (XLSX 12 KB)

Additional file 8: **Trans-eQTL loci results (for loci summarized in Table** 3 **).** Individual trans-eQTL loci results for those loci summarized in Table 3. (XLSX 32 KB)

Additional file 9: **Putative novel trans-eQTL and results at chr 11p15.5.** Putative novel trans-eQTL and results at chr 11p15.5. All cis and trans results for 11p15.5 are displayed. (XLSX 11 KB)

Additional file 10: **Long range cis eQTLs (P < 5E-8) and their short and long cis-eQTL associations.** Short- and long-range cis-eQTL associations for chromosome 16 and 20 regions with associations overlapping ENCODE 5C (chromatin conformation) interactions in lymphoblastoid cell lines. (XLSX 13 KB)

Additional file 11: **Significance of eSNPs relative to distance from their associated eGenes for different tissue types.** Significance of eSNPs relative to distance from their associated eGenes for different tissue types, respectively. PanelA: blood tissues and cell types (n = 14 datasets), PanelB: brain tissues (n = 24 datasets), PanelC: liver (n = 5 datasets), PanelD: fat-related (n = 3 datasets), PanelE: other tissues (n = 7 datasets). Y-axis is scaled to a cutoff at P < 1E-150 obscuring a small proportion of results. (DOC 1 MB)

Additional file 12: **cis-eQTL representation by chromosome (relative to length, gene #, RNA #, variation #).** Proportion of unique best cis- and trans-eQTLs by autosomal and sex chromosome. Proportions after adjustment for chromosome length, number of CCDS genes, total HuRef human RNA lengths, and number of HuRef variants are displayed, along with overall mean ranks for most to least *cis-*eQTLs per chromosomes across all adjustments. (XLSX 12 KB)

Additional file 13: **Comparison of eQTL results to NHGRI GWAS catalog SNPs.** Comparison of eQTL results (all or best eSNPs and their perfect proxies in HapMap CEU) to NHGRI GWAS catalog SNPs. (XLSX 9 KB)

Additional file 14: **Correlation between eQTL and GWAS p-values in the NHGRI GWAS catalog.** The correlation in strength of signal (represented by –log_10_ P-value) between reported eQTL studies and trait GWAS associations represented in the NHGRI GWAS catalog. (DOC 54 KB)

Additional file 15: **Enrichment or depletion of nSNPs (n = 100,601) and eQTLs (n = 62,872 best) among 45 full trait GWAS scans.** Pubmed identifiers and GWAS traits are given for 45 full GWAS scans whose results were compared to nSNPs (n = 100,601) and eQTLs (n = 62,872 best eSNPs). Genomic inflation factors (λ) are given for each trait and nSNPs and eQTLs for the full scans and at a threshold of P < 1E-2 in the GWAS. Komogorov-Smirnoff (K-S) test p-values for differences in distributions are given. Enrichments are highlighted in blue and depletions in grey, with significant K-S tests in red and non-significant ones in green. (XLSX 17 KB)

Additional file 16: **Kidney eQTLs reported in this study and association with GWAS traits (P < 5e-8).** Kidney eQTLs reported in this study were queries against the NHLBI GRASP GWAS database for overlaps. All GWAS intersections are given and GWAS results with particular relevance to renal function (serum creatinine, SLE and eGFR) are highlighted. (XLSX 13 KB)

Additional file 17: **Peripheral plaque eQTLs reported in this study and association with GWAS traits (P < 5e-8).** Plaque eQTLs reported in this study were queries against the NHLBI GRASP GWAS database for overlaps. All GWAS intersections are given and several associations with coronary artery disease and myocardial infarction are highlighted. (XLSX 38 KB)

Additional file 18: **Supplemental methods description of eQTL analysis for novel data (kidney, peripheral plaque, HBTRC brain).** Detailed methods and demographics for new eQTL analyses in included in this study. (DOCX 40 KB)

Additional file 19: **Flow chart of overall study, data collection and annotation and analysis.** Flow chart of overall study, data collection and annotation and analysis. (DOC 280 KB)
