# Supplementary material for: Synthesis of 53 tissue and cell line expression QTL datasets reveals master eQTLs
Source: BMC Genomics. 2014 Jun 27;15(1):532. doi: 10.1186/1471-2164-15-532 (PMC4102726; doi:10.1186/1471-2164-15-532)
Supplement: Supplementary file 3 — Additional file 3: Hierarchical clustering analysis of 248 eGenes found in ≥ 25/53 datasets used in pathway and ontology analyses. Clustering diagram of eGenes found in ≥ 25 datasets. (DOC 128 KB) [file 12864_2013_6258_MOESM3_ESM.doc]

**Supplementary Figure 1.** Hierarchical clustering analysis of 248 eGenes found in >= 25/53 datasets used in pathway and ontology analyses.

**248 eGenes in ≥ 25 eQTL datasets**
