# Supplementary material for: Synthesis of 53 tissue and cell line expression QTL datasets reveals master eQTLs
Source: BMC Genomics. 2014 Jun 27;15(1):532. doi: 10.1186/1471-2164-15-532 (PMC4102726; doi:10.1186/1471-2164-15-532)

**Supplementary Figure 2.** **Significance of eSNPs relative to distance from their associated eGenes for different tissue types, respectively.** 2A: blood tissues and cell types (n=14 datasets), 2B: brain tissues (n=24 datasets), 2C: liver (n=5 datasets), 2D: fat-related (n=3 datasets), 2E: other tissues (n=7 datasets). Y-axis is scaled to an cutoff at P<1E-150 obscuring a small proportion of results.

**Figure S2A. Blood tissue and cell line eQTL results (n=14 datasets).**

**
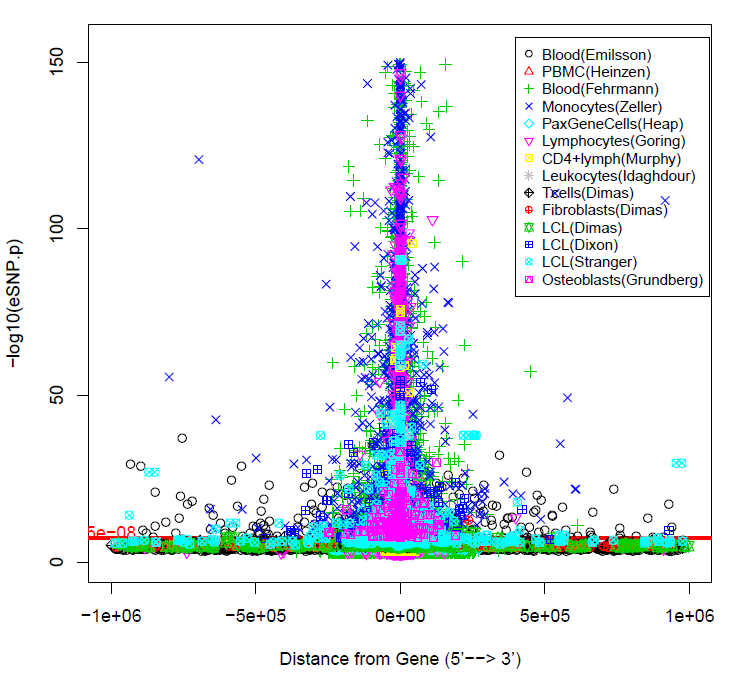
**

**Figure S2B. Brain tissue eQTL results (n=24 datasets).**

**
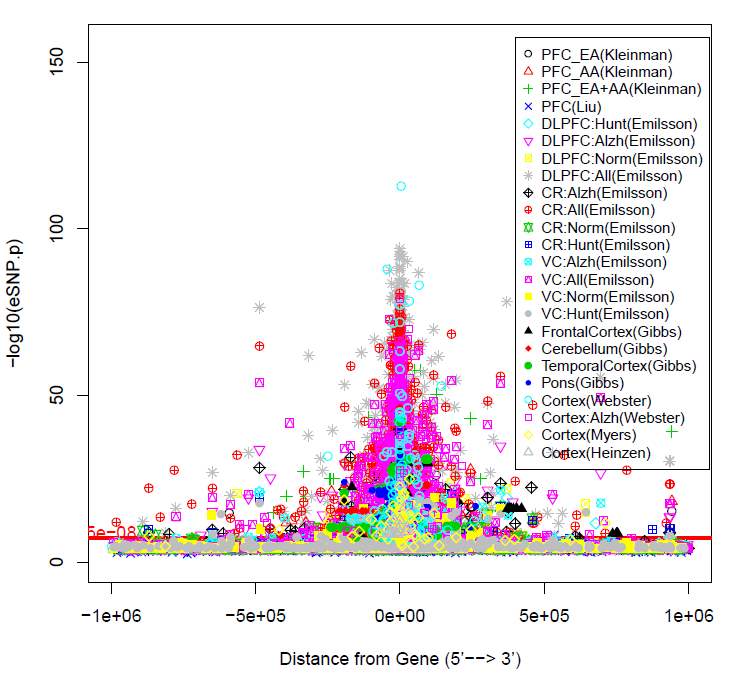
**

**Figure S2C. Liver *cis*-eQTL results (n=5 datasets).**


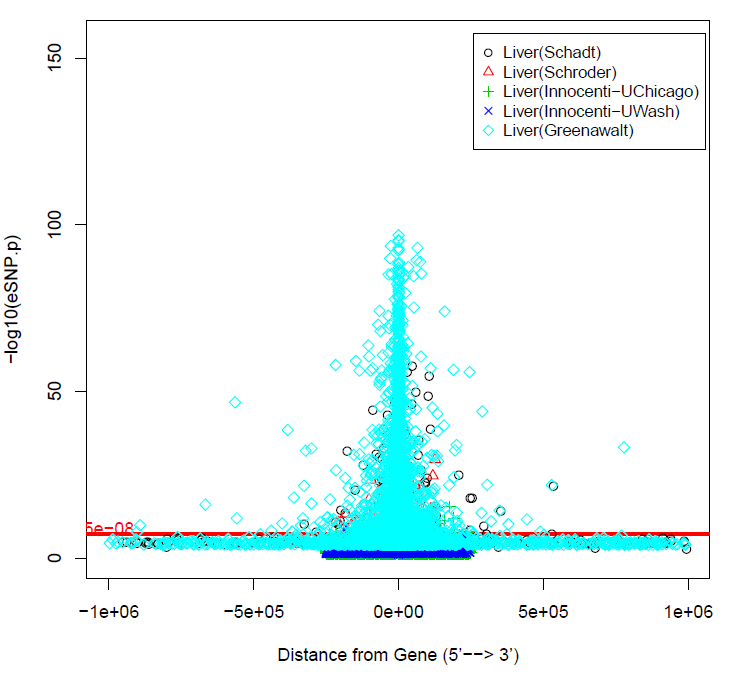


**Figure S2D. Adipose *cis*-eQTL results (n=3 datasets).**


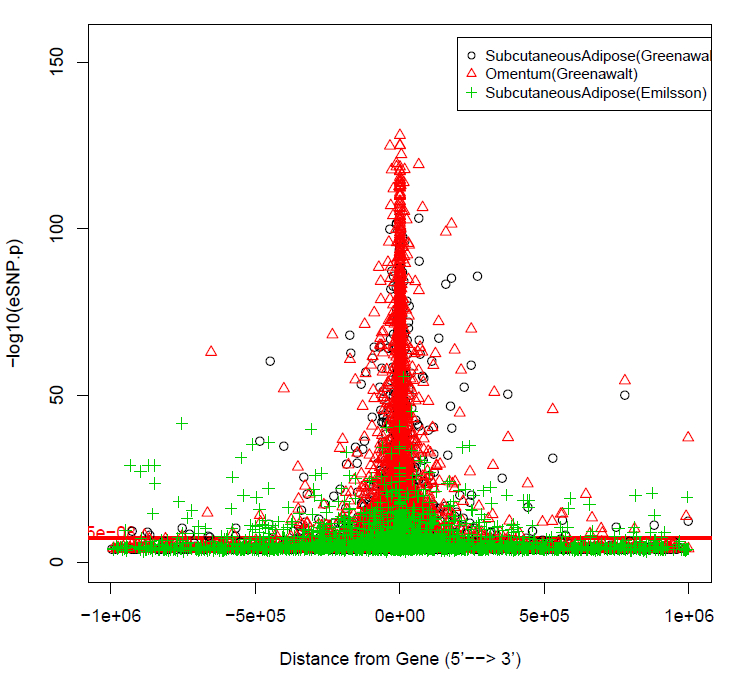


**Figure S2E. Other *cis*-eQTL results including skin, kidney, stomach, endometrial tumor and peripheral artery plaque (n=7 datasets).**


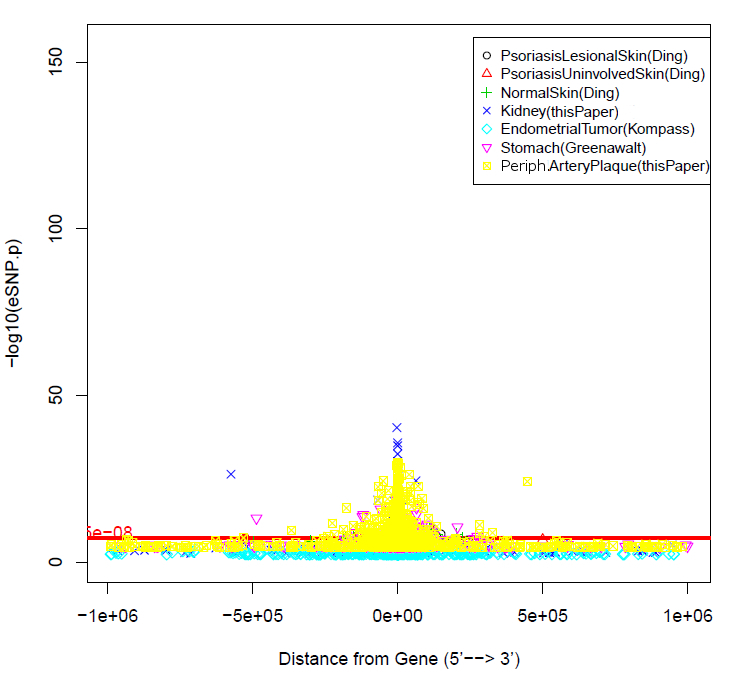

Supplement: Supplementary file 11 — Additional file 11: Significance of eSNPs relative to distance from their associated eGenes for different tissue types. Significance of eSNPs relative to distance from their associated eGenes for different tissue types, respectively. PanelA: blood tissues and cell types (n = 14 datasets), PanelB: brain tissues (n = 24 datasets), PanelC: liver (n = 5 datasets), PanelD: fat-related (n = 3 datasets), PanelE: other tissues (n = 7 datasets). Y-axis is scaled to a cutoff at P < 1E-150 obscuring a small proportion of results. (DOC 1 MB) [file 12864_2013_6258_MOESM11_ESM.doc]
