# Supplementary material for: Synthesis of 53 tissue and cell line expression QTL datasets reveals master eQTLs
Source: BMC Genomics. 2014 Jun 27;15(1):532. doi: 10.1186/1471-2164-15-532 (PMC4102726; doi:10.1186/1471-2164-15-532)
Supplement: Supplementary file 14 — Additional file 14: Correlation between eQTL and GWAS p-values in the NHGRI GWAS catalog. The correlation in strength of signal (represented by –log10 P-value) between reported eQTL studies and trait GWAS associations represented in the NHGRI GWAS catalog. (DOC 54 KB) [file 12864_2013_6258_MOESM14_ESM.doc]

**Supplementary Figure 3.** The correlation in strength of signal (represented by –log_10_ P-value) between reported eQTL studies and trait GWAS associations represented in the NHGRI GWAS catalog on 03-22-2013.
