# Supplementary material for: Synthesis of 53 tissue and cell line expression QTL datasets reveals master eQTLs
Source: BMC Genomics. 2014 Jun 27;15(1):532. doi: 10.1186/1471-2164-15-532 (PMC4102726; doi:10.1186/1471-2164-15-532)
Supplement: Supplementary file 18 — Additional file 18: Supplemental methods description of eQTL analysis for novel data (kidney, peripheral plaque, HBTRC brain). Detailed methods and demographics for new eQTL analyses in included in this study. (DOCX 40 KB) [file 12864_2013_6258_MOESM18_ESM.docx]

## Supplemental Methods Text to Zhang et al.

## HBTRC Expression & SNP Data Collection

1647 frozen tissue samples from cerebellum (CB), visual cortex (VC) BA17 and dorsolateral prefrontal cortex (PFC) BA9 were provided by the Harvard Brain Tissue Resource Center (HBTRC) at McLean Hospital (Belmont, MA). All autopsied brains, were collected from subjects with Alzheimer´s disease or Huntingdon’s disease diagnosis or from normal non-demented subjects, for whom both the donor and the next of kin had completed the HBTRC informed consent (http://www.brainbank). Tissue collection and the research were conducted according to the HBTRC guidelines (http://www.brainbank). The HBTRC samples were primarily of Caucasian ancestry, as only eight non-Caucasian outliers were identified and therefore excluded for further analysis. Post-mortem interval (PMI) was 17.8+8.3 hours, sample pH was 6.4±0.3 and RNA integrity number (RIN) was 6.8±0.8 for the average sample in the overall cohort. These were composed of 376 ALZHEIMER´S DISEASE patients, 193 HUNGTINDON’S DISEASE patients and 173 non-demented subjects.

RNA preparation and array hybridizations applied custom microarrays manufactured by Agilent Technologies consisting of 4,720 control probes and 39,579 probes targeting transcripts representing 25,242 known and 14,337 predicted genes. One *u*g of total RNA from each of the 2229 (ALZHEIMER´S DISEASE, non-demented, HD) brain tissues was reverse transcribed and labeled with either Cy3 or Cy5 fluorochrome. Purified Cy3 or Cy5 complementary RNA was hybridized to at least two single microarrays with fluor reversal for 24 hours in a hybridization chamber, washed, and scanned using a laser confocal scanner. Arrays were quantified on the basis of spot intensity relative to background, adjusted for experimental variation between arrays using average intensity over multiple channels, and fitted to an error model to determine significance (type I error) as previously described[19]. This microarray dataset is MIAME compliant and the raw gene expression data for all hybridizations together with information related to demographics, disease state (ALZHEIMER´S DISEASE or non-demented) and technical covariates (pH, RIN, PMI, batch, preservation method) are available via the GEO database (GEO accession number GSE44772 including the regional-specific subseries GSE44768, GSE44770, GSE44771).

Gene expression was reported as the mean-log ratio of individual microarray intensities relative to average intensities of all samples. Gene expression data were generated using Rosetta Resolver gene expression analysis software (version 7.0, Rosetta Biosoftware) and MATLAB (The MathWorks). To remove bias in expression profiles related to potential latent variables unrelated to underlying biological processes, we implemented a normalization method based on control probes present on the microarrays. We separated the control probes into two classes: (1) specialty probes, such as spike-in probes or other probes designed to monitor the quality of the microarrays; and (2) border probes used to describe the geometry of the microarray. We then identified the Principal Components (PC) explaining the variability of each control probe class and then identified the same components from a randomly permuted data set. We performed 10,000 permutations for each set of control probes and selected Principal Components with P-values defined as {number of [var(random-*PC_i_*) >var(*PC_i_*)]}/(number of permutations) < 10^−4^. The expression data in each tissue for each probe are thus the residuals from a linear model fitting incorporating the significant PCs.

We analyzed the contribution of each of the experimental covariate's age gender, sex and technical covariates. The Kolmogorov-Smirnov (KS) test was used to capture the difference of the correlation P-value distributions for each covariate per brain region and condition. Particular attention was paid to established covariates of mRNA quality that have been demonstrated in brain tissues, which included: (1) Extreme confounding of demographic variables in disease vs. controls; it was not possible to match samples based on demographic variables including age, sex, and ethnicity as many of these factors differ in their distributions substantially across the disease groups. Ethnicity was determined using STRUCTURE resulting in the exclusion of eight outliers (non-Caucasians), which were removed from all studies. (2) Pre-/post-mortem quantitative and quality factors including PMI, RIN, tissue pH status, manner of death and/or agonal state. Seven samples with RIN<5 were removed from the analysis. (3) Batch effects; day-to-day variability in the amplification process can influence the observed gene expression, even when profiled against a common-pool. For this particular data set, the batch effects are further exacerbated by the two additional facts: (i) the demographic compositions of the two batches differ, and (ii) for technical reasons, as it is preferable to have your pool sample be a representation of the greater population (mean-log ratio of individual microarray intensities was relative to average intensities of all samples in the final set). In summary, the distribution of P-values obtained from modeling the covariates listed above strongly suggest that adjusting for them would improve the downstream analysis steps. Therefore we chose a robust linear regression model for covariate corrections as rlm(expression~RIN + pH + PMI +age + batch + preservation + gender) and then the residuals were used for the genetics of gene expression analysis.

**HBTRC eQTL Detection**

Each of the 838,958 SNPs was tested for association to all 39,579 expression traits using Kruskal Wallis test based on the residuals, left after correction for covariates using robust linear regression as described above. Expression quantitative trait loci (eQTLs) for gene expression traits were determined by identifying the SNP most strongly associated with each expression trait profiled on the array over all the 838,958 genotyped SNPs. *Cis* analysis was limited to SNPs located within 1 Mb of either side of the transcription start or end within the gene body, while *trans* effects were defined as the associated SNPs located on a different chromosome to the physical location of the corresponding probe [19]. The association P-value was adjusted to control for testing of multiple SNPs and expression traits using two different methods: (1) a highly conservative Bonferroni correction method to constrain the study-wise significance level, and (2) an empirical FDR method [1] that constrains the overall rate of false positive events. For cis eQTL, to achieve a study-wise significance level of 0.05, the Bonferroni adjusted P-value threshold was computed as 0.05/(39,579×*N_i_*), where N_i_ denotes the number of SNPs tested for trait i within the two Mb window, over all 39,579 expression traits tested. The nominal P-value to meet this significance threshold is 3.0 × 10^−8^. The Bonferroni adjustment method can be conservative when there is dependence among the expression traits and among the SNP genotypes. Given that strong correlation structures exist among expression traits and among SNP genotypes in a given linkage disequilibrium (LD) block, the Bonferroni adjustment may be overly conservative. Therefore, we used an empirical FDR method based on permutations that accounts for the correlation structures among the expression traits and among the SNP genotypes. We constrained the empirically determined FDR to be less than 10%. Here, the FDR was estimated as the ratio of the average number of eQTLs found in datasets with randomized sample labels to the number of eQTLs identified in the original data set. Since the number of tests was large, we found the empirical null distribution was very stable and ten permutation runs were sufficient for convergence to estimate FDR. FDR computation was performed separately for cis and trans associations resulting in nominal P-value cutoffs of 5.0 × 10^−5^ and 1.0 × 10^−8^ for cis and trans eQTLs, respectively. The significance of the trans eQTL was also assessed by the Bonferroni method and by constraining the empirically determined FDR to be less than 10%. In the case of trans eQTL, all 838,958 SNPs were tested for association to each of the 39,280 expression traits. Therefore, the Bonferroni adjusted P-value threshold was computed as 0.05/(838,958×39,579) = 1.5 × 10^−12^.

cisTER summary

## cisTER Expression & SNP Data Collection

The FoxHollow SilverHawk Catheter device (ev3, Inc, Plymouth, MN; www.ev3.net) was used to obtain 290 peripheral plaque from the common femoral, superficial femoral, anterior tibial, peroneal, popliteal, posterior tibial, or tibial peroneal trunk arteries as previously described[2]. RNA was successfully isolated from 202 peripheral plaque samples and profiled on a custom Affymetrix whole-genome expression array platform RM-HU01Aa520485 containing 43,737 features corresponding to 39,003 unique accession numbers throughout the genome. Expression data was normalized using the standard RMA method [3]. Genotyping was performed on Affymetrix 6.0 Genome-wide chips. SNP probe sets were called with Affymetrix GTC Software. As a quality control all samples were checked for sex-specific probes on both RM-HU01Aa520485 and 6.0 Genome-wide chip data. All SNP probe sets with <90% calls and minor allele frequency (MAF)<5% were removed, that resulted in a total of 224,698 SNPs used in the analysis.

**cisTER eQTL Detection**

To find the cis- and trans-acting expression quantitative trait loci (eSNPs/eQTLs) we used a method based on a previous publication [15]. Briefly though, the Kruskal-Wallis test was used to determine association between adjusted expression traits and the genotypes. cis eQTLs for gene expression traits were determined by identifying the SNP most strongly associated with each expression trait profiled on the array over all genotyped SNPs typed within 1 Mb of the probe of interest. Results are reported at a 10% false discovery rate (FDR) based on permutations of the association between SNP genotypes and expression levels for any given expression trait. Trans eQTLs were determined using a similar approach, but instead considering all SNPs >1 Mb from the probe of interest. Representative eSNPs for each trans eQTL were then selected as the most statistically significantly associated SNP within the eQTL region.

Kidney eQTL summary

**Kidney expression & SNP Data Collection**

All samples for each cohort were analyzed on Affymetrix U133 A&B set. Expression was normalized within each cohort using dChip (perfect match probe sets only). Genotyping was performed on Affymetrix 6.0 Genome-wide chips. SNP probe sets were called with Affymetrix GTC Software. As a quality control all samples were checked for sex-specific probes on both U133 and 6.0 Genome-wide chip data. The overall average SNP probe call rate was 98%. For both cohorts, all SNP probe sets with <90% calls were removed.

**Kidney gene and SNP annotation**

We used Perl and R scripts to link every SNP probe set to the nearest upstream and downstream genes using the mapped RefSeq annotation from the Affymetrix annotation files (build 30). To preserve potential isoform specific eQTLs, all combinations of SNP-expression probe sets were then linked to the annotated RefSeqs per expression probe set using Affymetrix annotation (build 30). In total, 29,782 unique RefSeq annotations map to 18,930 unique genes. In total there are over 3 million eQTL combinations of SNP probe sets linked to a unique RefSeq linking to a unique expression probe set.

**Kidney multivariate regression**

To find eQTLs, we used R scripts to perform a linear multivariate regression (see equation below) within each cohort, where Yij = base 2 logarithm of normalized expression for the U133 probe set of SNP(j) in kidney sample (i). Only cortex samples were used, so tissue was not a variable. Parameters: g=genotype; age = age ; anc = race (Caucasian or other); s= sex (male or female). Eij is a random error term. Coefficients β(1 to 4) are estimated by least squares.

$$Y\text{ij}=\beta\text{0j}+\beta\text{1j}g\text{ij}+\beta\text{2j}age\text{i}+\beta\text{3j}anc\text{i}+\beta\text{4j}s\text{i}+\varepsilon\text{ij}$$

**Fisher’s Combined Probability Test**

We used R scripts and Bioconductor to calculate a meta-Pvalue over both cohorts using a Fisher’s combined probability test. Only those eQTL combinations with a nominal P<0.05 (for genotype) and a Beta in the same direction in both cohorts were selected, yielding 9989 eQTL combinations (Meta-P value range: 1.7e-02 to 2.75e-35). For each independent P value (p) of test (i), they are combined into one test statistic (X^2^). X^2^ follows a chi-square distribution with 2k degrees of freedom, (k) being the number of tests being combined.

$$X^{2}=-2\sum_{i=1}^{k} log\text{e}\left( p\text{i} \right)$$

**Kidney Permutation analysis**

To determine the False Discovery Rate (FDR) of our eQTL data, we used R scripts to perform a permutation. We performed a label swap for the samples (to preserve linkage disequilibrium between SNPs). 1000 permutations on each cohort were seeded randomly using the Stanford BioX2 supercluster with a LSF batch system. A combined P value for each seed was calculated using Fisher’s combined test (see previous paragraph). The FDR cutoff of Q<0.025 was iterated for the true dataset: At a cutoff Pvalue of 2.90e-5; FDR is 0.025 (i.e. the average nr. of permuted eQTLs is 31 (peak at 28) which is 2.5% of the nr. of true eQTLs for that cutoff (1220).

**Kidney Pairwise LD analysis**

SNAP LD [41] was used to prune the eQTL SNPs for the minimal set of unlinked independent eQTLs, using the linkage disequilibrium (LD) data from HapMap release 22, CEU population panel. The R^2^ threshold used was 0.5 and a maximal distance of 500kb. The pairwise LD was calculated between all SNPs within 500 kb distance of each other and all linked SNPs were but one from each LD block, retaining the SNP with the lowest Pvalue.

**Baseline characteristics of the kidney samples for the eQTL analysis.**

|  | **Cohort 1** | **Cohort 2** |
| --- | --- | --- |
| **Sample size, n** | 56 | 25 |
| **Age, mean (range)** | 65 (29–87) | 62 (36–84) |
| **Males, n ( %)** | 32 (57%) | 18 (72%) |
| **Caucasians, n (%)** | 43 (76%) | 16 (64%) |

In total we had 81 biopsies of normal kidney cortex tissue from transplantation donors or nephrectomy patients [4,5]. We genotyped 2 independent cohorts of unrelated subjects for which we had gene expression data from kidney cortex samples: Cohort 1 - gene expression data from [4], and Cohort 2 - gene expression data from [5].

Age, sex and race were all variables in the multivariate regression (see methods), hence all reported eQTLs had a significant effect for genotype independent of age, sex or race.

References

1. Storey JD, Tibshirani R (2003) Statistical significance for genomewide studies. Proc Natl Acad Sci U S A 100: 9440-9445. 10.1073/pnas.1530509100 [doi];1530509100 [pii].

2. Puig O, Yuan J, Stepaniants S, Zieba R, Zycband E, Morris M, Coulter S, Yu X, Menke J, Woods J, Chen F, Ramey DR, He X, O'Neill EA, Hailman E, Johns DG, Hubbard BK, Yee LP, Wright SD, Desouza MM, Plump A, Reiser V (2011) A gene expression signature that classifies human atherosclerotic plaque by relative inflammation status. Circ Cardiovasc Genet 4: 595-604. CIRCGENETICS.111.960773 [pii];10.1161/CIRCGENETICS.111.960773 [doi].

3. Irizarry RA, Hobbs B, Collin F, Beazer-Barclay YD, Antonellis KJ, Scherf U, Speed TP (2003) Exploration, normalization, and summaries of high density oligonucleotide array probe level data. Biostatistics 4: 249-264. 10.1093/biostatistics/4.2.249 [doi];4/2/249 [pii].

4. Rodwell GE, Sonu R, Zahn JM, Lund J, Wilhelmy J, Wang L, Xiao W, Mindrinos M, Crane E, Segal E, Myers BD, Brooks JD, Davis RW, Higgins J, Owen AB, Kim SK (2004) A transcriptional profile of aging in the human kidney. PLoS Biol 2: e427. 10.1371/journal.pbio.0020427 [doi].

5. Wheeler HE, Metter EJ, Tanaka T, Absher D, Higgins J, Zahn JM, Wilhelmy J, Davis RW, Singleton A, Myers RM, Ferrucci L, Kim SK (2009) Sequential use of transcriptional profiling, expression quantitative trait mapping, and gene association implicates MMP20 in human kidney aging. PLoS Genet 5: e1000685. 10.1371/journal.pgen.1000685 [doi].
