# Supplementary material for: Synthesis of 53 tissue and cell line expression QTL datasets reveals master eQTLs
Source: BMC Genomics. 2014 Jun 27;15(1):532. doi: 10.1186/1471-2164-15-532 (PMC4102726; doi:10.1186/1471-2164-15-532)

**Supplementary Figure 3.** Flow chart of overall study, data collection and annotation and analysis.


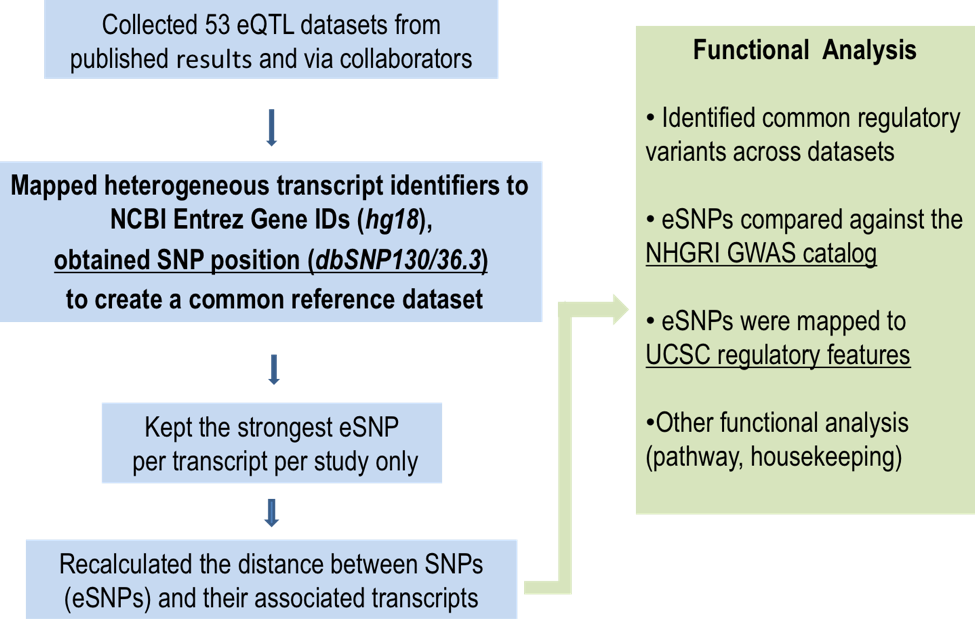

Supplement: Supplementary file 19 — Additional file 19: Flow chart of overall study, data collection and annotation and analysis. Flow chart of overall study, data collection and annotation and analysis. (DOC 280 KB) [file 12864_2013_6258_MOESM19_ESM.doc]
